# Androgen-Induced Cell Migration: Role of Androgen Receptor/Filamin A Association

**DOI:** 10.1371/journal.pone.0017218

**Published:** 2011-02-16

**Authors:** Gabriella Castoria, Loredana D'Amato, Alessandra Ciociola, Pia Giovannelli, Tiziana Giraldi, Leandra Sepe, Giovanni Paolella, Maria Vittoria Barone, Antimo Migliaccio, Ferdinando Auricchio

**Affiliations:** 1 Dipartimento di Patologia Generale, II Università di Napoli, Napoli, Italy; 2 European Laboratory for the Investigation of Food Induced Disease, Dipartimento di Pediatria, Università ‘Federico II’, Napoli, Italy; 3 Dipartimento di Biochimica e Biotecnologie Mediche, Università ‘Federico II’, Napoli, Italy; Florida International University, United States of America

## Abstract

**Background:**

Androgen receptor (AR) controls male morphogenesis, gametogenesis and prostate growth as well as development of prostate cancer. These findings support a role for AR in cell migration and invasiveness. However, the molecular mechanism involved in AR-mediated cell migration still remains elusive.

**Methodology/Principal Findings:**

Mouse embryo NIH3T3 fibroblasts and highly metastatic human fibrosarcoma HT1080 cells harbor low levels of transcriptionally incompetent AR. We now report that, through extra nuclear action, AR triggers migration of both cell types upon stimulation with physiological concentrations of the androgen R1881. We analyzed the initial events leading to androgen-induced cell migration and observed that challenging NIH3T3 cells with 10 nM R1881 rapidly induces interaction of AR with filamin A (FlnA) at cytoskeleton. AR/FlnA complex recruits integrin beta 1, thus activating its dependent cascade. Silencing of AR, FlnA and integrin beta 1 shows that this ternary complex controls focal adhesion kinase (FAK), paxillin and Rac, thereby driving cell migration. FAK-null fibroblasts migrate poorly and Rac inhibition by EHT impairs motility of androgen-treated NIH3T3 cells. Interestingly, FAK and Rac activation by androgens are independent of each other. Findings in human fibrosarcoma HT1080 cells strengthen the role of Rac in androgen signaling. The Rac inhibitor significantly impairs androgen-induced migration in these cells. A mutant AR, deleted of the sequence interacting with FlnA, fails to mediate FAK activation and paxillin tyrosine phosphorylation in androgen-stimulated cells, further reinforcing the role of AR/FlnA interaction in androgen-mediated motility.

**Conclusions/Significance:**

The present report, for the first time, indicates that the extra nuclear AR/FlnA/integrin beta 1 complex is the key by which androgen activates signaling leading to cell migration. Assembly of this ternary complex may control organ development and prostate cancer metastasis.

## Introduction

AR controls morphogenesis, gametogenesis and prostate growth at puberty. It also represents a hallmark of prostate cancer in adults. These processes occur through a reciprocal interplay between epithelial and mesenchymal cells. Urogenital sinus mesenchyme induces, for instance, development and differentiation of epithelial cells that in turn control differentiation and localization of mesenchymal progenitors. Again, proper development of the genital tubercle and the embryonic anlage of external genitalia in the male phenotype also require coordinated outgrowth of the mesodermally-derived mesenchyme and extension of the endodermal urethra within an ectodermal epithelial capsule. The re-awakening of mesenchymal interactions are involved in benign prostate hyperplasia and prostate cancer in adults [1]. Taken together, this evidence raises the question as to whether androgens and AR directly control mesenchymal cells as well as their migratory phenotype.

Fibroblasts and keratinocytes express AR during wound healing [2], and stromal AR may play opposite roles in different stages of prostate cancer, either blocking or promoting prostate cancer metastasis [3]. We previously reported that NIH3T3 fibroblasts and mouse embryo fibroblasts (MEFs) harbor classical AR [4]. In NIH3T3 cells, this receptor is localized outside the nucleus and does not activate gene transcription. It does, nevertheless, activate various signaling effectors and triggers different phenotypes depending on hormone concentration. At suboptimal R1881 concentration (1 pM), AR induces S-phase entry through recruitment and activation of Src as well as phosphatidylinositol-3-kinase (PI3-K; proliferative phenotype). At a more physiological androgen concentration (10 nM), AR only slightly increases S-phase entry, whereas it triggers Rac 1 activation and cytoskeleton changes (migratory phenotype) in the absence of association with Src and PI3-K [4]. This suggests that AR associates with other effectors to regulate cell migration.

Physiological androgen concentration (10 nM) also induces G0/G1 arrest and cytoskeleton changes in human fibrosarcoma HT1080 cells engineered to stably express high levels of AR [5]. AR knockout retards neutrophil maturation and reduces their migration rate [6]. These findings support a role for AR in cell migration and invasiveness. However, they do not address the molecular mechanism underlying these processes.

The filamin (Fln) family consists of three homologous proteins, A, B and C. Mouse models of Fln deficiency have underlined the regulatory role of these proteins in cell migration [7]. Indeed, mutations in Fln genes (A and B) cause a wide range of brain, bone, skeleton and heart developmental malformations, likely due to severe defects in embryonic cell migration as well as to the failure of Fln to interact with other proteins [8]. FlnA and its proteolytic fragments directly interact with AR, thereby modulating nuclear translocation and transcriptional action of AR as well as androgen dependence of prostate cancer LNCaP cells [9], [10], [11]. Further, cytoplasmic localization of FlnA has been correlated with metastatic and hormone-refractory phenotypes in human prostate cancer, suggesting that intracellular localization of FlnA orchestrates invasiveness and even hormone responsiveness of prostate cancers [12]. FlnA also recruits and activates the downstream effector of GTPases, PAK-1 [13]. Rapid activation of this kinase by estradiol has been reported in breast cancer cells and has been correlated to modifications in shape and polarity of these cells [14]. Remarkably, the non-genomic activation of PAK-1 by estradiol controls hormone negative feedback actions in the reproductive axis *in vivo* [15]. In sum, FlnA intersects steroid action at different levels and in different cellular compartments by directly anchoring steroid receptors (eg AR) or signaling effectors (GTPases and their upstream or downstream molecules) that mediate rapid steroid effects.

Here, we report that 10 nM R1881 significantly enhances association and co-localization of AR with FlnA at cytoskeleton of NIH3T3 fibroblasts. This complex also recruits integrin beta 1. The androgen-driven assembly of AR/FlnA/integrin beta 1 complex induces activation of Rac 1 and FAK, which both coordinate migration. Results with human fibrosarcoma HT1080 cells strengthen the significance of these findings. Like NIH3T3 cells, HT1080 cells harbor low levels of AR and undergo cell migration upon androgen stimulation. The present findings provide new clues explaining the regulatory role of FlnA in androgen-regulated cell motility in mesenchymal cells and reveal a novel and unexpected role for AR/FlnA interaction in the extra nuclear compartment of cells. This complex acts as a linker between androgen signaling and actin cytoskeleton, thereby driving cell migration. It may affect human organ development as well as cancer progression and could thus offer a new target to gain a more tailored therapy of androgen-dependent human cancers.

## Results

### Androgen induces migration of NIH3T3 fibroblasts

**Figure 1:**
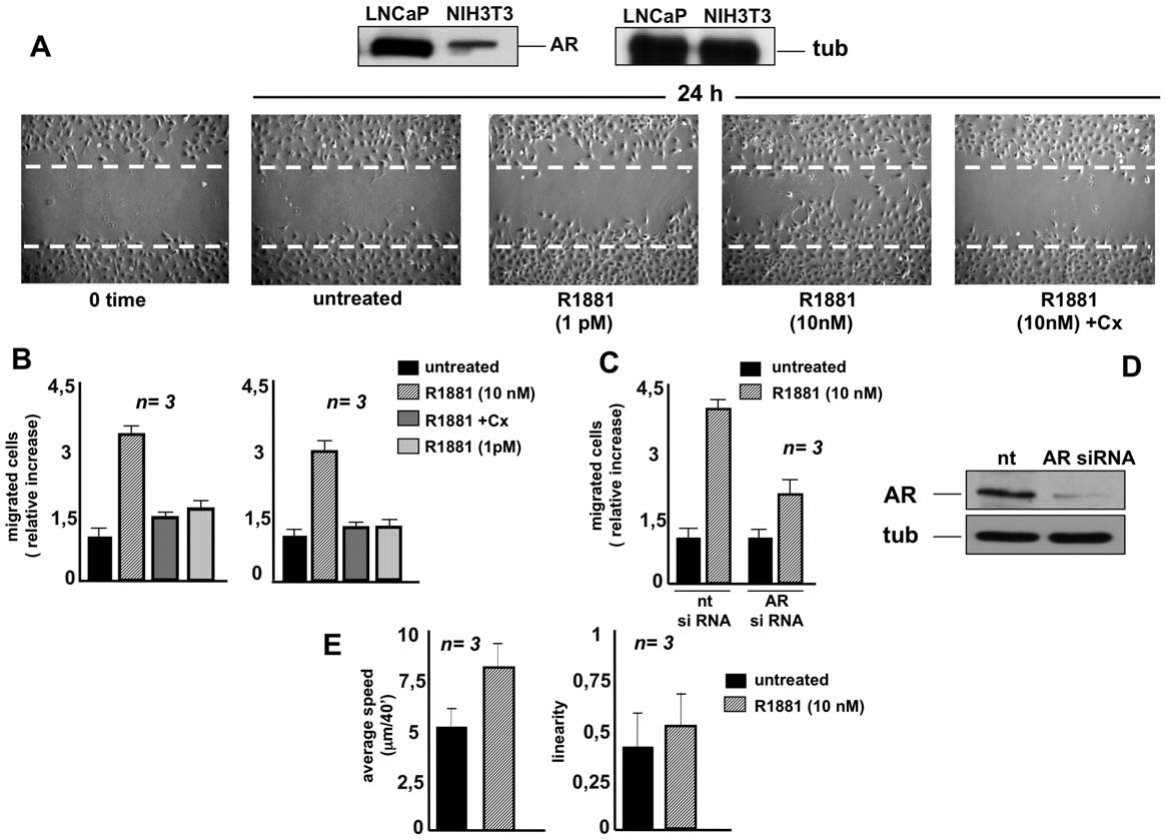
R1881 stimulates migration in NIH3T3 fibroblasts. Quiescent NIH3T3 cells were used. In **A,** upper panels: Western blot of lysate proteins from NIH3T3 and prostate cancer-derived LNCaP cells with anti-AR (left) or anti-tubulin (right) antibodies. In **A**, lower panels: NIH3T3 cells were wounded and then left unstimulated or stimulated for the indicated time with R1881 (at 10 nM or 1 pM). When indicated, Casodex (Cx) was added at a 1000-fold excess. Contrast-phase images are representative of four different experiments, each performed in duplicate. In **B,** migration assay on collagen-coated (left graph) or uncoated (right graph) Transwell filters was performed in the absence or presence of the indicated compounds. Casodex (Cx) was added at a 1000-fold excess. Migrated cells were stained as described in Methods and data were expressed as relative increase in number of migrated cells. In **C** and **D**, growing NIH3T3 cells were transfected with siRNA Alexa Fluor 488 along with either control siRNA (nt) or AR siRNA. After transfection, the cells were made quiescent. In **C**, the cells were allowed to migrate in collagen-coated Transwell filters in the absence or presence of 10 nM R1881. Migrated cells were stained as described in Methods and data were expressed as relative increase in number of migrated cells. In **D,** lysate proteins were immunoblotted using the antibodies against the indicated proteins (androgen receptor, AR; tubulin, tub). In **E,** video time-lapse microscopy was followed in the absence or presence of 10 nM R1881. Data were analyzed as described in Methods and cell motility expressed as average speed (microm/40′; left graph). In both experimental conditions, no significant influence in linearity of cell motility was detected (right graph). In **B**, **C** and **E,** means and SEM are shown; ***n*** represents the number of experiments. The statistical significance of results in **B**, **C** and **E** was also evaluated by Student's *t* test. In **B** (left and right panels), P values were <0.005 for cells stimulated with 10 nM R1881. No significance was attributed to the difference in relative migration between unstimulated cells and cells stimulated with 10 nM R1881 in the presence of Casodex. Again, no significance was attributed to the difference in relative migration between unstimulated cells and cells stimulated with 1 pM R1881. In **C**, the difference in relative migration between cells transfected with AR siRNA and those transfected with control siRNA and challenged with 10 nM R1881 was significant (P<0.001). Also significant (P<0.001) was the difference in relative migration between cells transfected with control siRNA and left unstimulated or stimulated with 10 nM R1881. In **E**, the difference in cell motility between unstimulated cells and cells stimulated with 10 nM R1881 was significant (P<0.005).

NIH3T3 cells harbor a very low amount of AR as compared to that expressed in prostate cancer-derived LNCaP cells (upper panel in Fig. 1A) and [4]. Upon 10 nM R1881 stimulation of NIH3T3 cells, this receptor rapidly activates Rac 1 thereby promoting cytoskeleton changes [4]. To determine whether these changes trigger cell migration, quiescent NIH3T3 cells were wounded and allowed to migrate in the absence or presence of the compounds indicated in Fig. 1A (lower panels). Contrast-phase images show that wound area was significantly reduced in cells treated with 10 nM R1881. In contrast, only few cells migrated upon treatment with a very low R1881 concentration (1 pM), which induces a proliferative phenotype [4]. Addition of the anti-androgen Casodex consistently inhibited the 10 nM androgen-induced effect, indicating that classical AR is involved in this response. Control images captured at 0-time or from untreated cells are shown for comparison. Neither estradiol nor progestin R5020 affected migration of NIH3T3 cells, which do not express estradiol receptor alpha (ER alpha) or progesterone receptor (PR; Fig. S1) and [4]. These results further confirm that AR mediates androgen stimulation of NIH3T3 cell migration.

Consistent with findings in Fig. 1A, panel B shows that stimulation of quiescent NIH3T3 cells with 10 nM R1881 increases by 3-fold the number of cells migrated through collagen-coated (left graph) or uncoated (right graph) Transwell filters. Similar data were obtained using 10 nM androgen dihydrotestosterone (DHT, not shown). A negligible effect was observed in cells challenged with low R1881 concentration (1 pM). Here again, the 10 nM R1881-induced effect was significantly reduced by addition of the androgen antagonist Casodex, thus supporting the view that classical AR mediates the observed effects. This issue was conclusively addressed by the experiment in panel C showing that AR knockdown markedly inhibits the number of migrated cells upon androgen stimulation. The Western blot in Fig. 1D shows that AR expression is almost completely abolished by siRNA (upper panel) in the presence of equal amounts of loaded tubulin (lower panel).

Finally, NIH3T3 cells were followed in video time-lapse microscopy for 24 h and migration was quantitatively evaluated by recording the paths followed by each cell. Fig. 1E (left graph) shows that treatment of NIH3T3 fibroblasts with 10 nM R1881 increases the average speed from about 5 to 8 µm/40 min, as measured by averaging all cell displacements. The effect on motility speed is consistently present during the whole observation time, as demonstrated by averaging cell displacements at each time step (not shown). Path linearity was also evaluated as the ratio between net displacement and path length for each observed cell. Right graph in Fig. 1E shows that R1881 fails to produce any significant effects. It also indicates that the various cell populations show similar distributions. Thus, 10 nM R1881 affects migration speed without altering migration type.

In conclusion, data in Fig. 1 show that 10 nM R1881 enhances migration of NIH3T3 fibroblasts through classical AR.

### Androgen modifies cytoskeleton and increases focal adhesions as well as tyrosine phosphorylation of FAK and paxillin in NIH3T3 fibroblasts

Changes in the cytoskeleton architecture and tyrosine phosphorylation of proteins at focal contact sites are hallmarks of migrating cells. In a preliminary experiment, we analyzed actin remodeling upon androgen treatment of NIH3T3 cells in a time course experiment (Fig. S2). Ten nM R1881 treatment induces membrane ruffling and protrusions in NIH3T3 fibroblasts within 20 minutes. These changes are dynamically shut off within 1 h (Fig. S2) and [10]. A more prolonged stimulation with 10 nM R1881 slightly affects the actin organization (Fig. S2). These latter findings do not exclude, however, that very rapid and periodic waves of actin remodeling occur to accomplish cell migration [16], [17].

**Figure 2:**
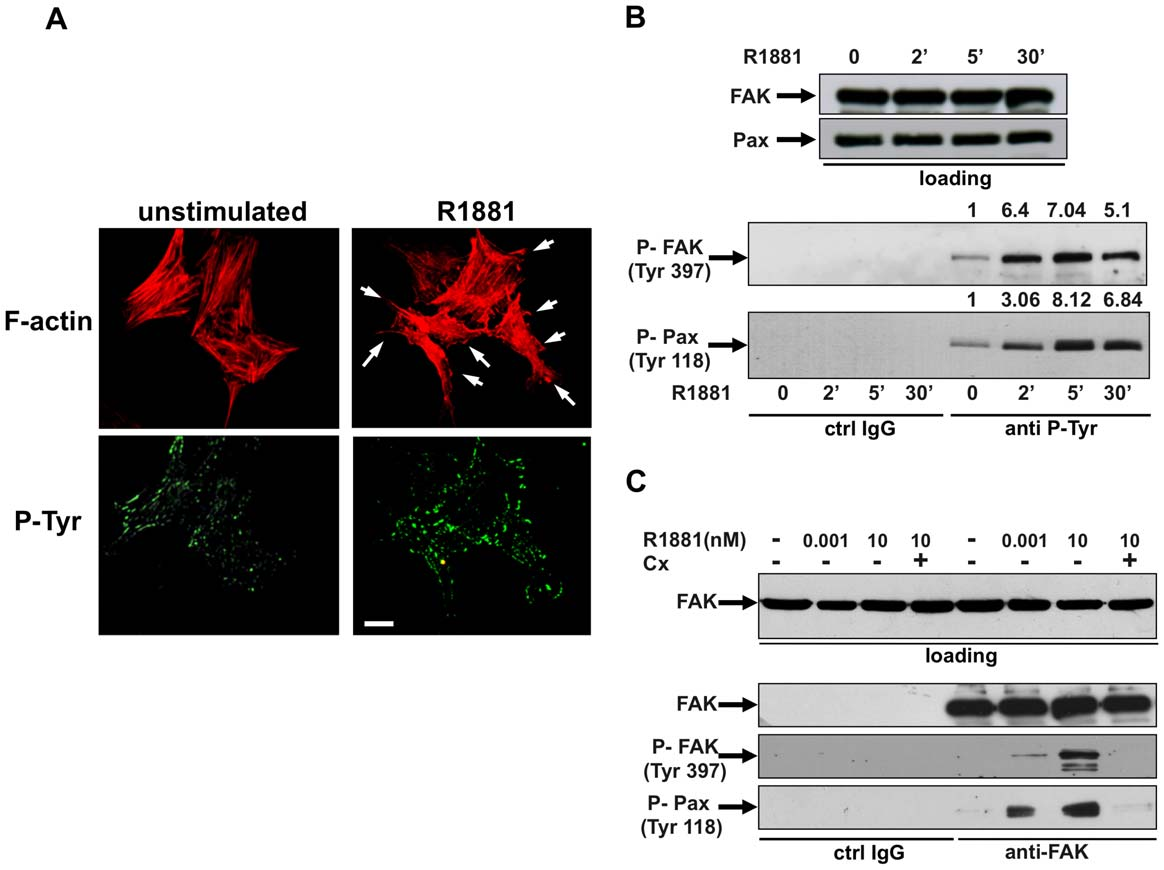
R1881-stimulated fibroblast migration: the role of FAK and paxillin. In **A,** **B** and **C**, quiescent NIH3T3 cells were used. In **A**, cells on coverslips were left unstimulated or stimulated for 20 min with 10 nM R1881 and then analyzed by IF for F-actin (upper images) and P-Tyr (lower images). Images are representative of three independent experiments. Arrows indicate the protrusions and ruffles caused by androgen treatment of cells. Scale bar, 5 microm. In **B** and **C,** cells were untreated or treated for the indicated times (**B**) or for 5 min (**C**) with R1881 (at 1 pM or 10 nM). Casodex (Cx) was added at a 1000-fold excess. In **B**, cell lysates were immunoblotted using antibodies against FAK or paxillin (loading). Similar amounts of lysate proteins were immunoprecipitated with control IgG (from Pierce; ctrl IgG) or anti-P-Tyr (anti-P-Tyr) antibody. Immunoprecipitated proteins were analyzed using antibodies against the indicated proteins. For each phospho-blot, data were analyzed using the NIH Image J program and expressed as relative increase. Numbers in the upper portion of corresponding blots represent the increase in P-FAK or P-paxillin. In **C**, cell lysates containing similar amounts of total FAK (loading) were incubated with control IgG (ctrl IgG) or anti-FAK antibody and proteins in immunocomplexes were blotted using antibodies against the indicated proteins.

We then investigated by immunofluorescence (IF) analysis the effect of androgens on actin remodeling and P-Tyr distribution in NIH3T3 cells. Fig. 2A shows that 10 nM R1881 treatment causes ruffles and protrusions (upper panels) within 20 min, together with a simultaneous and significant increase in P-Tyr spots, associated with the ends of actin stress fibers at the cell periphery of NIH3T3 cells (lower panels). Since tyrosine phosphorylation of FAK and the focal contact-associated protein paxillin is involved in cell adhesion and motility [18], [19], we analyzed the effect of androgen treatment on FAK and paxillin tyrosine phosphorylation. Data in Fig. 2B show that 10 nM R1881 stimulation rapidly triggers FAK Tyr 397 phosphorylation in lysates from NIH3T3 cells immunoprecipitated with anti-P-Tyr antibody (lower panel). This effect is maximal at 5 min and declines at 30 min of hormonal stimulation. Tyr 118-phosphorylated paxillin parallels tyrosine phosphorylation of FAK upon androgen stimulation of cells (lower panel in Fig. 2B). The pull-down is specific since neither P-Tyr FAK nor P-Tyr paxillin were detected in lysates immunoprecipitated with IgG antibody, used as a control. Again, upper panels in B show that similar amounts of total FAK and paxillin were loaded in immunoprecipitation experiments. The effect we observed is androgen-specific, since neither estradiol (Fig. S3, panel A) nor progestin R5020 (Fig. S3, panel B) affect FAK or paxillin tyrosine phosphorylation. Finally, using anti-FAK antibody in an immunoprecipitation experiment we observed that 10 nM R1881 stimulation of quiescent NIH3T3 cells markedly increases P-Tyr 397-FAK and P-Tyr 118-paxillin (Fig. 2C). The anti-androgen Casodex abolishes androgen-induced tyrosine phosphorylation of FAK and paxillin. In cells stimulated with 1 pM R1881, which only slightly stimulates cell migration, a weak increase in both P-Tyr 397-FAK and P-Tyr 118-paxillin was observed (Fig. 2C). Specificity of immunoprecipitation is confirmed by the absence of FAK or P-Tyr 397 FAK or P-Tyr 118 paxillin in lysates immunoprecipitated with a control IgG antibody.

**Figure 3:**
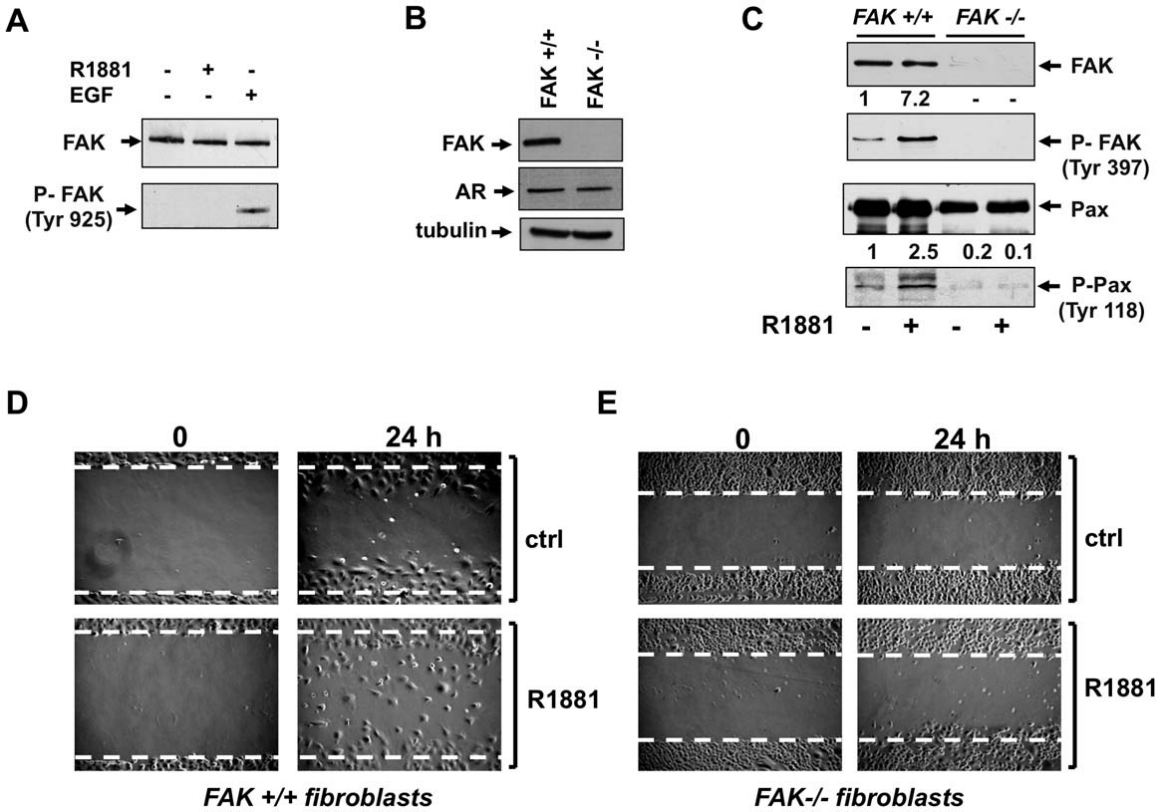
R1881-stimulated fibroblast migration: the role of FAK. In **A,** quiescent NIH3T3 cells were used. Cells were untreated or treated for 5 min with R1881 (at 10 nM) or EGF (at 100 ng/ml; from Roche). Cell lysates were immunoblotted using antibodies against FAK or P-Tyr 925 FAK. In **B**, **C**, **D** and **E**, quiescent FAK+/+ or FAK-/- fibroblasts were used. In **B**, cell lysates were analyzed by Western blot using antibodies against FAK, AR or tubulin. In **C**, cells were untreated or treated for 5 min with 10 nM R1881 and lysates were immunoblotted with antibodies against the indicated proteins. For each phospho-blot, data were analyzed using the NIH Image J program and expressed as relative increase. Numbers in the upper portion of corresponding blots represent the increase in P-Tyr 397 FAK or P-Tyr 118 paxillin. In **D** and **E**, the cells were wounded and left unstimulated or stimulated with 10 nM R1881. The cells were then allowed to migrate for 24 h. Contrast-phase images are representative of three different experiments, each performed in duplicate.

Since FAK activity is also regulated by phosphorylation at Tyr 925 [19], we analyzed by Western blot the effect of androgens on this phosphorylation. Data in Fig. 3A shows that stimulation of NIH3T3 fibroblasts with 10 nM R1881 does not trigger P-Tyr 925-FAK phosphorylation. In contrast, EGF treatment of cells markedly increases such a phosphorylation. These findings indicate that in our experimental conditions androgen-induced cell migration does not involve FAK phosphorylation at Tyr 925.

### Androgen-induced cell migration: the role of FAK

To further analyze the role of FAK in androgen-induced cell migration, we used FAK+/+ and FAK-/- fibroblasts. The Western blot in Fig. 3B confirms that FAK-/- fibroblasts do not express FAK (upper panel), while they still express AR (middle panel). The lower panel shows that similar amounts of lysate proteins were loaded. Stimulation of FAK+/+ fibroblasts with 10 nM R1881 induces strong tyrosine phosphorylation of FAK (upper panels in C) and paxillin (lower panels in C), thus indicating that paxillin tyrosine phosphorylation is controlled by FAK activity in androgen-treated cells. In contrast, 10 nM R1881 fails to induce both FAK and paxillin tyrosine phosphorylation in FAK-/- fibroblasts (upper and lower panels in C).

Finally, both FAK+/+ as well as FAK-/- fibroblasts were wounded and allowed to migrate in the absence or presence of 10 nM R1881. Contrast-phase images in Fig. 3D show that in FAK+/+ fibroblasts, a significant number of cells move towards the wound area upon challenging with 10 nM R1881. In contrast, androgens do not stimulate migration of FAK-/- fibroblasts (panel E).

Altogether, findings in Fig. 2 and 3 show that FAK contributes to androgen-induced cell migration and that FAK activation controls paxillin tyrosine phosphorylation in androgen-treated cells.

### Androgen-induced cell migration: the role of Rac 1

Ten nM R1881 triggers rapid Rac 1 activation in parallel with cytoskeleton changes in NIH3T3 fibroblasts [10]. We therefore addressed the upstream events regulating Rac 1 activation as well as the role of Rac in androgen-induced cell motility of NIH3T3 fibroblasts.

**Figure 4:**
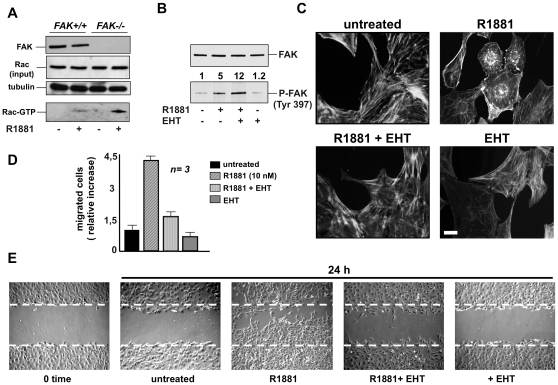
R1881-stimulated fibroblast migration: the role of Rac. In **A,** quiescent FAK+/+ or FAK-/- fibroblasts were used. Cells were then left unstimulated or stimulated for 5 min with 10 nM R1881 and lysate proteins were analyzed by immunoblot using anti-FAK (FAK), Rac (input) or tubulin antibody. Rac activity in cell lysates was assayed in pull-down experiments (Rac-GTP). Data were analyzed using the NIH Image J program. In androgen-treated FAK-null fibroblasts, the relative increase in Rac-GTP was 3.7-fold compared to the androgen-treated FAK+/+ fibroblasts. In **B, C,** **D** and **E**, quiescent NIH3T3 cells were used. In **B**, cells were left unstimulated or stimulated for 5 min with 10 nM R1881 in the absence or presence of EHT (20 microM). Control cells were treated with EHT alone. Lysates were analyzed by immunoblot using anti-P-Tyr 397 FAK (P-Tyr-FAK) or FAK (FAK) antibodies. Data were analyzed using the NIH Image J program and expressed as relative increase. Numbers in the upper portion of corresponding blots represent the increase in P-FAK. In **C**, cells on coverslips were left untreated or treated for 40 min with the indicated compounds. R1881 was used at 10 nM and EHT 1864 at 20 µM. Cells were then analyzed by IF for F-actin. Images are representative of three independent experiments. Scale bar, 5 microm. In **D,** migration assay on collagen-coated Transwell filters was performed in the absence or presence of the indicated compounds. R1881 was used at 10 nM and EHT 1864 at 20 microM. Migrating cells were stained with Hoechst and counted with fluorescent microscope as described in Methods. Data were expressed as relative increase in number of migrated cells. Means and SEM are shown; ***n*** represents the number of experiments. The statistical significance of results was also evaluated by Student's *t* test. P values were <0.001 for cells stimulated with 10 nM R1881. The difference in relative migration between cells challenged with 10 nM R1881 in the absence or presence of EHT was significant (P<0.001). The difference in relative migration between unstimulated cells and cells stimulated with 10 nM R1881 in the presence of EHT or between unstimulated cells and EHT-treated cells was not significant. In **E**, the cells were wounded and then left unstimulated or stimulated for the indicated time with 10 nM R1881 in the absence or presence of 20 microM EHT. Control cells were treated with EHT alone (at 20 microM). Contrast-phase images are representative of three different experiments, each performed in duplicate.

Various mechanisms regulate the timing of activation of Rho family proteins. Among these, FAK-dependent activation is certainly involved [20]. We therefore analyzed by pull-down assay the activation of Rac 1 in FAK+/+ and FAK-/- fibroblasts. Fig. 4A shows that 10 nM R1881 stimulation activates Rac 1 in both FAK+/+ and FAK-/- fibroblasts. These findings exclude the possibility that androgen-dependent Rac activation depends on FAK. Although similar amounts of Rac 1 were loaded, androgen-induced Rac 1 activation is even more evident in FAK-/- fibroblasts (Fig. 4A). This finding might be related to the dual role of FAK, which acts as both a signaling kinase and as an adaptor/scaffold protein. Thus, in FAK-/- fibroblasts the absence of FAK association with regulators of Rho family proteins might enable the increase in androgen-induced Rac activation here observed.

In turn, it has been reported that Rac is an upstream regulator of FAK in Schwann cell motility induced by IGF-1 [21]. We therefore analyzed the role of Rac in androgen-induced FAK activation. The Western blot analysis in Fig. 4B shows that androgen significantly increases FAK auto-phosphorylation. Addition of the Rac inhibitor HT1864 even enhances the androgen effect, while EHT1846 alone does not significantly modify the basal level of FAK p-Tyr phosphorylation. Altogether, these findings support the view that FAK activation does not depend on Rac and vice versa in NIH3T3 cells stimulated with androgens.

Various signaling effectors, including Rho GTPase family members, Akt and MAPKs, play a role in cell migration [20]. We therefore analyzed the effect of 10 nM R1881 on activation of these effectors in NIH3T3 fibroblasts. Fig. S4 shows that no change in Rho A activity was observed in pull-down assay of androgen-treated NIH3T3 cell lysates (panel A). Again, 10 nM R1881 had no effect on the activation of Akt (Fig. S4, panel B) or extracellular-regulated kinase (Erk; Fig. S4, panel C). These findings do not support the hypothesis that Rho, Akt or Erk activation is involved in androgen-regulated cell migration in these cells.

We therefore analyzed the role of Rac activation in regulation of actin cytoskeleton and migration induced by androgens in NIH3T3 cells. Fig. 4C shows that the Rac inhibitor EHT1846 greatly reduces cytoskeleton changes caused by 10 nM R1881 treatment of NIH3T3 cells. Consistently, EHT1864 inhibits androgen-induced cell migration in both Transwell (Fig. 4D) and wound scratch (Fig. 4E) assays.

Findings in Fig. 4 show that androgen-induced cell migration involves Rac. The results also indicate that FAK and Rac are independently activated and that both converge on cell migration once activated.

### Androgens induce cell migration in human fibrosarcoma cells: the role of Rac 1

**Figure 5:**
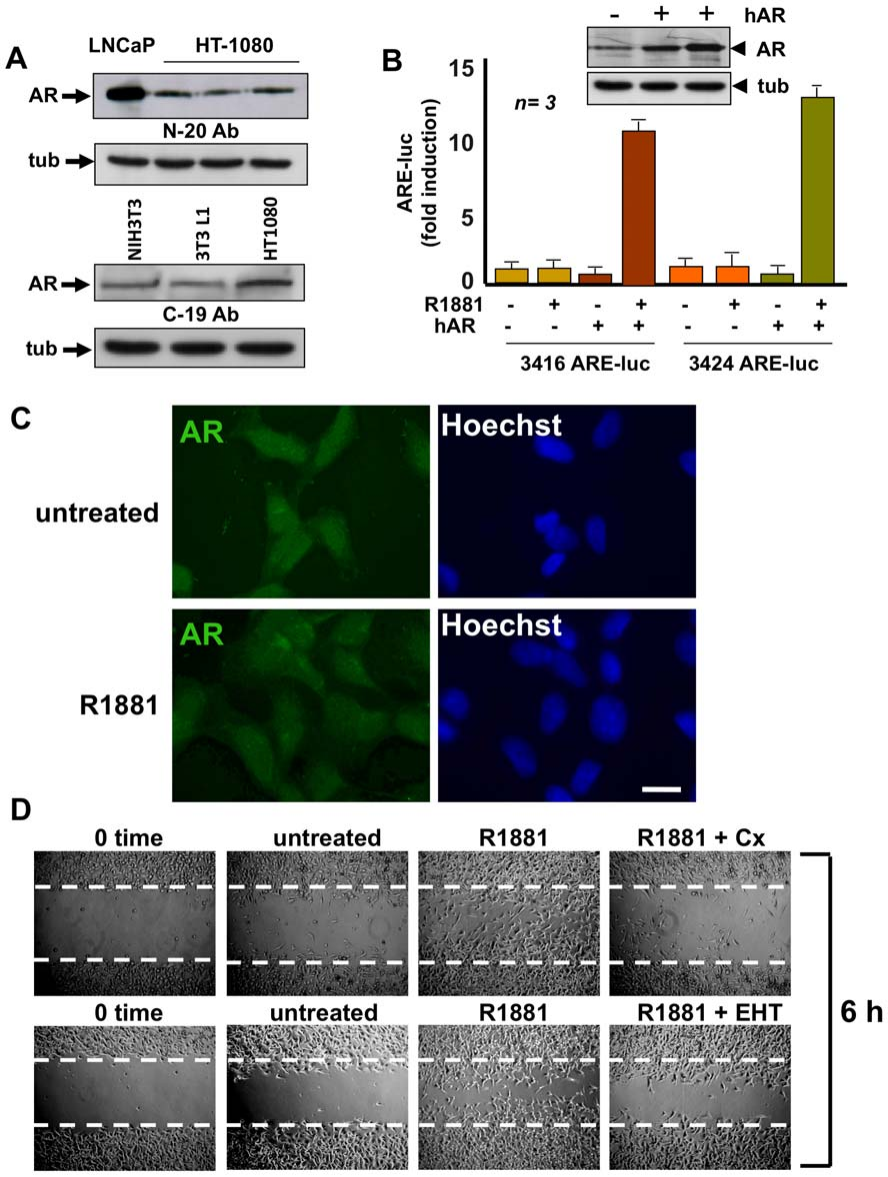
Human fibrosarcoma HT1080 cells harbor transcriptionally inactive AR that stimulates cell migration through androgen activation of Rac. Quiescent fibrosarcoma HT1080 cells were used. Panel **A** shows the Western blot of cell lysates with the N-20 (upper) or C-19 (lower) anti-AR antibody. Immunoblot analysis of lysates from growing LNCaP or NIH3T3 or 3T3-L1 cells was performed for comparison. In each panel, the levels of tubulin (tub) were determined by Western blot. In **B**, the cells were transfected with either 3416 or 3424 ARE-luc constructs in the absence or presence of human AR (hAR) expressing plasmid. Cells were made quiescent and then left unstimulated or stimulated for 18 h with 10 nM R1881. Luciferase activity was assayed, normalized using beta-gal as an internal control, and expressed as fold induction. Data from three independent experiments were analyzed. Means and SEM are shown; ***n*** represents the number of experiments. The statistical significance of results was also evaluated by Student's *t* test. In cells co-transfected with hAR and 3416 ARE-luc, the difference in ARE-luc induction between cells challenged with 10 nM R1881 and unstimulated cells was significant (P<0.001). Also significant (P<0.001) was the difference in ARE-luc induction between cells co-transfected with hAR and 3424 ARE-luc and left unstimulated or challenged with 10 nM R1881. Over-expression of hAR in HT1080 cells was verified by Western blot analysis using the C-19 anti-AR antibody (upper inset in **B**). The levels of tubulin (tub) were also determined (lower inset in **B**). In **C**, cells on coverslips were left untreated or treated for 60 min with 10 nM R1881 and then analyzed by IF for AR (left) or nuclei (Hoechst; right). Images are representative of three independent experiments. Scale bar, 5 microM. In **D**, the cells were wounded, then left unstimulated or stimulated for the indicated time with 10 nM R1881. Casodex (Cx) was used at 1000-fold excess and EHT 1864 at 20 microM. Contrast-phase images are representative of three different experiments.

We investigate the effect of androgen in cell migration of human fibrosarcoma HT1080 cells. The upper panel in Fig. 5A shows that HT1080 cells express a low level of AR compared to that expressed in LNCaP cells. It should be noted that expression of AR is also much lower in NIH3T3 cells than in LNCaP cells (Fig. 1) and [4]. AR expression was also detected by immunoblot of HT1080 cell lysates with an antibody directed at COOH-terminal sequence of AR (Fig. 5A, lower panel).

We then assessed by gene reporter assay the transcriptional activity of AR in HT1080 cells. Fig. 5B shows that HT1080 cells harbor a transcriptionally incompetent AR, as assayed using two different ARE-reporter genes. This failure to induce gene transcription is not due to the cellular milieu, since over-expression of wt human AR (hAR) enables these cells to efficiently activate transcription upon androgen stimulation. The inset in panel B confirms that wt hAR is actually over-expressed in HT1080 cells.

We then surveyed by IF analysis the effect of androgens on AR nuclear translocation. Panel C shows that under basal conditions, AR is equally distributed between nucleus and cytoplasm of quiescent HT1080 cells. One-hour stimulation of cells with 10 nM R1881 failed to induce AR nuclear translocation and a similarly diffuse distribution of AR was observed at later time points (2 and 4 h; not shown). In contrast, images captured from prostate cancer-derived LNCaP cells or Cos cells ectopically expressing wt hAR show that 10 nM R1881 clearly induces nuclear translocation of AR (Fig. S5).

Finally, quiescent HT1080 cells were wounded and allowed to migrate in the absence or presence of 10 nM R1881. Images in Fig. 5D (upper panels) show that wound area is markedly reduced in cells treated with 10 nM R1881. Control images captured at 0-time or from untreated cells are shown for comparison. The anti-androgen Casodex and the Rac inhibitor EHT1864 consistently inhibit androgen-induced migration (Fig. 5D, lower panels). These results show that through its receptor androgens also induce the migratory phenotype in fibrosarcoma cells, further supporting the role of Rac in this phenotype.

### Androgen-triggered AR association with FlnA and integrin beta 1 leads to FAK activation

In the search for a link between AR, focal contact sites and cytoskeleton proteins, we reasoned that FlnA, an actin-binding protein, might be involved. This hypothesis was based on the well-known role of FlnA in cell motility [22] as well as the reported interaction between AR and FlnA proteolytic peptides in various cell types, including LNCaP cells [9], [10].

**Figure 6:**
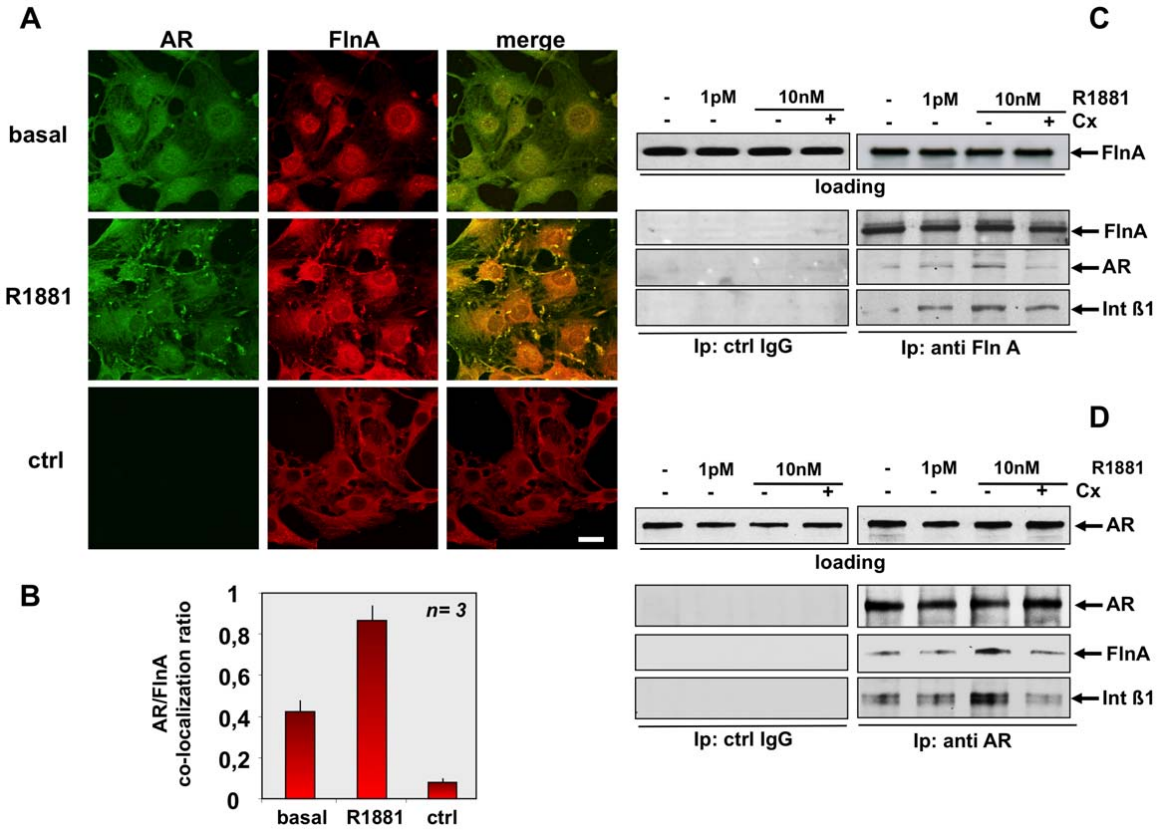
R1881-induced AR/FlnA/integrin beta 1 complex in fibroblasts. Quiescent NIH3T3 cells were left untreated (basal) or treated for 5 min with 10 nM R1881 (R1881). In **A**, cells on coverslips were visualized by IF for AR and FlnA. Images captured by confocal microscope show the staining of AR (green) and FlnA (red). Right panels show the merged images. The lowest images are representative of a control IF staining devoid of the primary anti-AR antibody (ctrl). Left image shows FITC-secondary antibody alone, middle image shows Fln A (red). Right image shows the merge. Scale bar, 5 microM. Graph in panel **B** represents the quantification and statistical analysis of different experiments. AR/FlnA co-localization ratio was calculated as described in Methods. Means and SEM are shown; ***n*** represents the number of experiments. The statistical significance of results was also evaluated by Student's *t* test. The difference in AR/FlnA co-localization ratio between unstimulated (basal) and R1881-stimulated cells was significant (P<0.005). Also significant (P<0.005) was the difference in AR/FlnA co-localization ratio between control cells (ctrl) and either unstimulated (basal) or R1881-stimulated cells. In **C** and **D**, quiescent NIH3T3 cells were left untreated or treated for 5 min with the indicated concentrations of R1881. The anti-androgen Casodex (Cx) was used at 10 microM. Cell lysates were immunoblotted with antibody against the indicated proteins (loading). Lysate proteins containing similar amounts of FlnA (**C**) or AR (**D**) were immunoprecipitated with anti-FlnA (**C**) or anti-AR (**D**) antibody. Similar amounts of lysate proteins were also immunoprecipitated with control IgG (ctrl IgG). Proteins in immunocomplexes were analyzed using antibodies against the indicated proteins.

Confocal microscopy analysis in Fig. 6A shows that challenging quiescent NIH3T3 cells with 10 nM R1881 increases the co-localization between AR/Fln A at cytoskeleton within 5 min. Control images (ctrl) were captured from cells stained with the FITC-conjugated secondary antibody alone, as a control. They show that AR staining is specific. Quantification and statistical analysis of results obtained from three independent experiments is presented in Fig. 6B.

FlnA directly interacts with the cytoplasmic domain of integrin beta 1 and mediates actin-dependent processes [23]. We therefore verified by immunoprecipitation experiments whether androgens trigger a complex assembly made up of FlnA, AR and integrin beta 1 in NIH3T3 cells. The immunoprecipitation experiment in Fig. 6C shows that stimulation of NIH3T3 cells with 10 nM R1881 induces recruitment of AR and integrin beta 1 to FlnA. Again, stimulation with 1 pM R1881 increases association of integrin beta 1 with FlnA, but fails to stimulate recruitment of AR to this complex at levels comparable to those observed at 10 nM R1881. Notably, Casodex prevents the 10 nM R1881-induced association of AR with FlnA and integrin beta 1. Neither FlnA, nor AR, nor integrin beta 1 were detected in lysates immunoprecipitated with anti-IgG antibody, used as a control.

In a reciprocal immunoprecipitation experiment we observed that stimulation of NIH3T3 cells with 10 nM R1881 induces recruitment of integrin beta 1 and FlnA to AR specifically immunoprecipitated from lysates of NIH3T3 cells (Fig. 6D). Again, stimulation with 1 pM R1881 fails to stimulate recruitment of integrin beta 1 and FlnA to AR. Likewise, the complex assembly induced by 10 nM R1881 was blocked by Casodex. Neither AR, nor FlnA, nor integrin beta 1 were detected in lysates immunoprecipitated with IgG antibody, used as a control (Fig. 6D). Because of the role of integrin beta 3 in filamin signaling [24], we also explored whether this integrin participates in androgen signaling. Ten nM R1881 increases association of AR with FlnA, but fails to induce recruitment of integrin beta 3 to this complex in NIH3T3 cells (Fig. S6). Expectedly, Casodex prevents the 10 nM R1881-induced association of AR with FlnA. Regardless of experimental conditions, a small amount of integrin beta 3 was detected in a co-immunoprecipitation experiment (Fig. S6).

**Figure 7:**
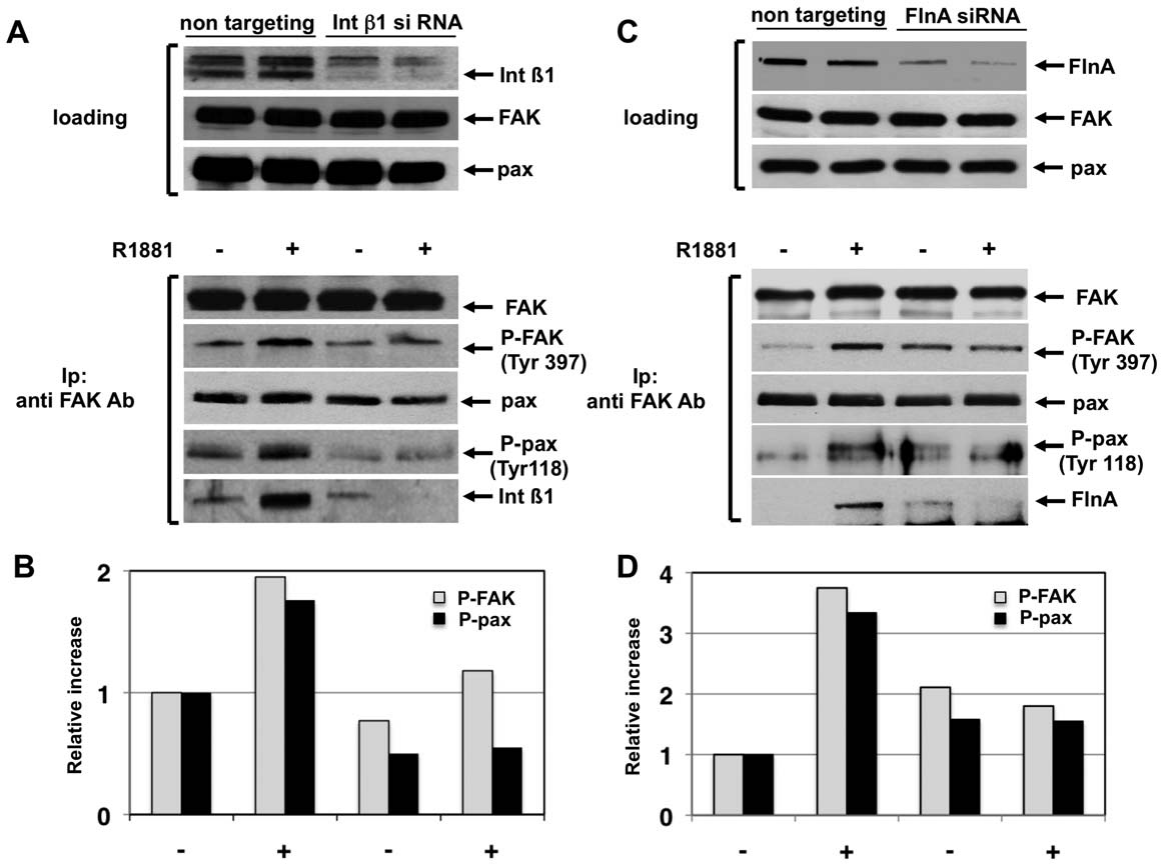
R1881-induced AR/FlnA/integrin beta 1 complex controls FAK activation and tyrosine paxillin phosphorylation in fibroblasts. Growing NIH3T3 cells were transfected with control siRNA (non-targeting) or integrin beta 1 siRNA (**A**) or FlnA siRNA (**C**) as described in Methods. After transfection, cells were made quiescent and then untreated or treated for 5 min with 10 nM R1881. Upper sections show the Western blot of corresponding cell lysates with anti-integrin beta 1 (**A**) or FlnA (**C**) antibodies. The levels of FAK and paxillin were also assessed by Western blot. Lysate proteins were immunoprecipitated with anti-FAK antibody (lower sections in **A** and **C**) and proteins in immunocomplexes were analyzed by Western blot using the antibodies against the indicated proteins. The increase in P-FAK and P-paxillin was analyzed using the NIH Image J program and results are graphically expressed as relative increase. The graph in **B** refers to the experiment in **A**, whereas the graph in **D** refers to the experiment in **C**.

Finally, the role of FlnA/AR/integrin beta 1 complex in androgen-induced effects on focal complexes was addressed by siRNA experiments. Fig. 7A shows that knockdown of integrin beta 1 abolishes the 10 nM R1881-induced P-Tyr 397-FAK phosphorylation as well as P-Tyr 118 phosphorylation of paxillin in lysates immunoprecipitated with anti-FAK antibody. In control cells, transfected with non-targeting RNA, a weak co-immunoprecipitation of integrin beta 1 with FAK was observed under basal conditions. This association was significantly stimulated by androgens. In all experimental conditions, similar amounts of total paxillin were detected (Fig. 7A). Lastly, quantification of P- FAK and P-paxillin phosphorylation under the different experimental settings was calculated and graphically shown (Fig. 7B).

Silencing of FlnA abolishes the 10 nM R1881-induced P-Tyr 397-FAK phosphorylation as well as P-Tyr 118 phosphorylation of paxillin in lysates immunoprecipitated with anti-FAK antibody (Fig. 7C). In cells transfected with non-targeting RNA, FlnA does not co-immunoprecipitate with FAK under basal conditions. Androgen treatment of cells induces this association and stimulates recruitment of FlnA to FAK (Fig. 7C). Here again, no change in the total amount of paxillin was detected (Fig. 7C). Quantification of P-FAK and P-paxillin phosphorylation under the different experimental conditions was also calculated and graphically shown (Fig. 7D).

**Figure 8:**
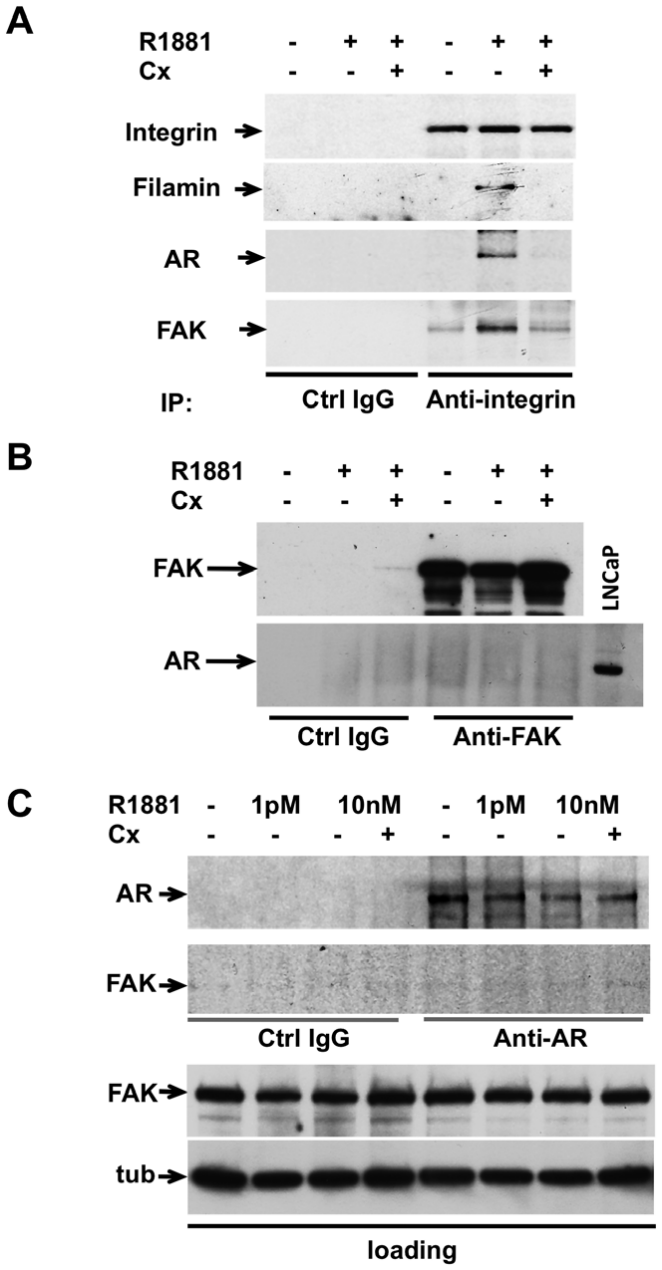
R1881 induces AR/FlnA/integrin beta 1/FAK complex in fibroblasts. Quiescent NIH3T3 cells were used. In **A**, cells were left untreated or treated for 5 min with 10 nM R1881 (R1881) in the absence or presence of 10 µM Casodex (Cx). Lysate proteins were immunoprecipitated with either control (Ctrl IgG) or anti-integrin beta 1 antibody. In **B,** cells were left untreated or treated for 5 min with 10 nM R1881 (R1881) in the absence or presence of 10 microM Casodex (Cx). Lysate proteins were immunoprecipitated with either control (Ctrl IgG) or anti-FAK antibody. In **C**, cells were left untreated or treated for 5 min with R1881 (1 pM or 10 nM) in the absence or presence of 10 microM Casodex (Cx). Lysate proteins containing similar amounts of FAK or tubulin (loading) were immunoprecipitated with either control (Ctrl IgG) or anti-AR antibody. In **A**, **B** and **C**, proteins in immunocomplexes were analyzed by immunoblotting using antibodies against the indicated proteins. When indicated, lysate proteins from LNCaP cells were analyzed for AR expression by immunoblotting with anti-AR antibody.

By using anti-integrin beta 1 antibody we detected co-immunoprecipitation of FlnA/AR/FAK in lysates from NIH3T3 cells challenged with 10 nM R1881. Addition of Casodex to the cell medium prevented the 10 nM R1881-induced FlnA/AR/FAK complex assembly. Neither FlnA, nor AR, nor FAK were detected in lysates immunoprecipitated with anti-IgG antibody, used as a control (Fig. 8A). Under the same experimental conditions, we did not detect AR/FAK complex in lysates from NIH3T3 fibroblasts immunoprecipitated with either anti-FAK (Fig. 8B) or anti-AR (Fig. 8C) antibody. In addition, confocal microscopy analysis did not show AR/FAK co-localization in androgen-treated NIH3T3 cells (Fig. S7). Collectively, these data make unlikely a direct association between AR and FAK. Rather, they suggest that upon androgen challenging the AR/FlnA-associated integrins signal to other integrin molecules that in turn recruit FAK, triggering its auto-phosphorylation. Therefore, the failure to detect AR/FAK association by co-immunoprecipitation might be due to a high dissociation rate of these large protein complexes. However, other possibilities cannot be ruled out.

Taken together, results in Figs. 6, 7 and 8 support each other in indicating that androgens challenge an AR/FlnA/integrin beta 1 complex outside cell nucleus. This complex controls FAK auto-phosphorylation, which s poised downstream of the AR/FlnA/integrin beta 1 complex assembly.

### AR/FlnA complex is required for androgen-induced cell migration as well as Rac 1 activation

**Figure 9:**
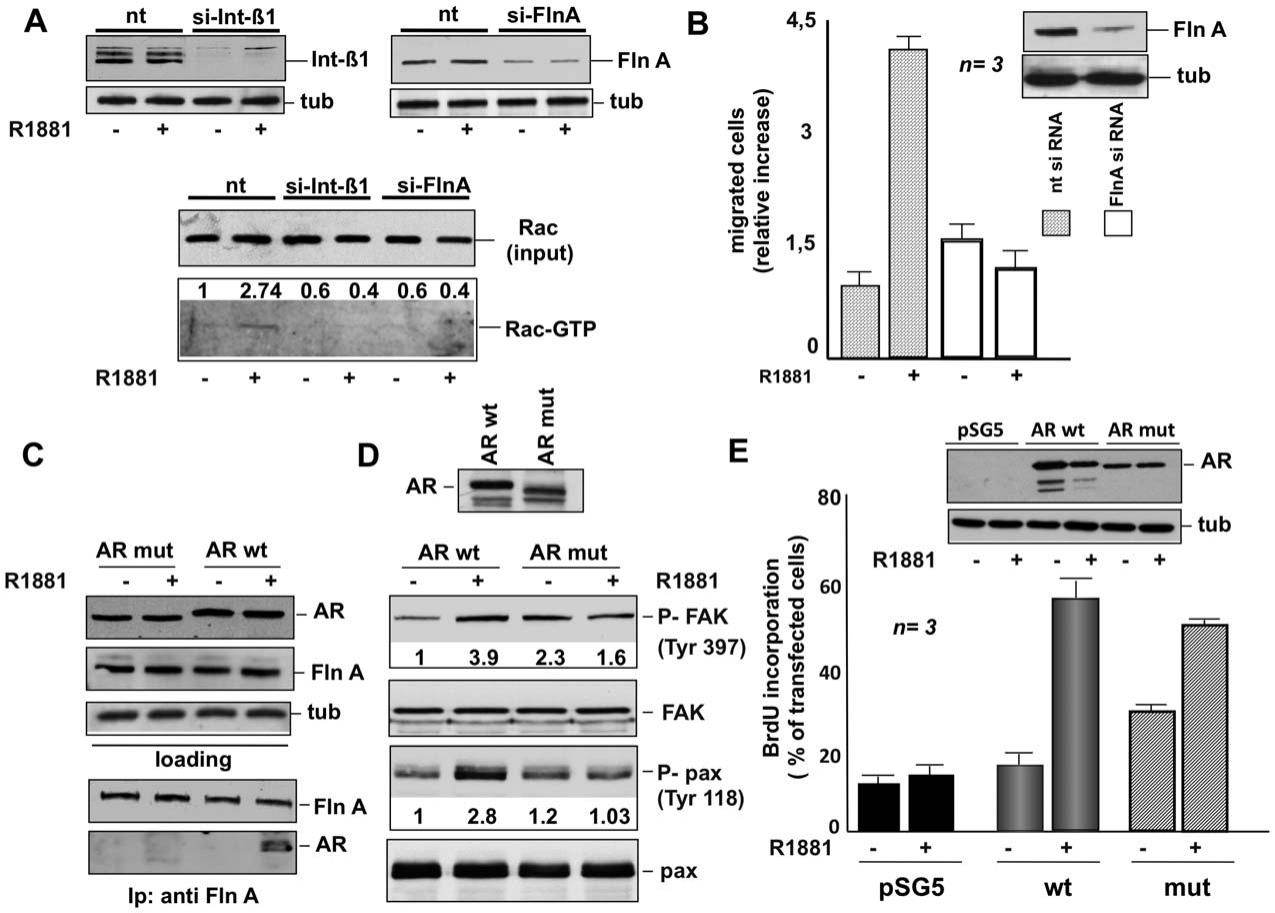
Role of AR, FlnA and integrin beta 1 in androgen-triggered activation of signaling responsible for fibroblast migration. In **A**, growing NIH3T3 cells were transfected with control siRNA (nt) or integrin beta 1 siRNA or FlnA siRNA as described in Methods. After transfection, the cells were made quiescent and then left untreated or treated for 5 min with 10 nM R1881. The upper section shows the Western blot of corresponding cell lysates with anti-integrin beta 1 (left) or FlnA (right) antibody. The levels of tubulin were also assessed by Western blot (tub). Cell lysates containing similar amounts of total Rac (input) were used to assay Rac activation in pull-down experiments (Rac-GTP). Data were analyzed using the NIH Image J program and expressed as relative increase. Numbers in the upper portion of corresponding blots represent the increase in Rac-GTP. In **B**, growing NIH3T3 cells were transfected with siRNA Alexa Fluor 488 along with either control siRNA (nt) or FlnA siRNA. The cells were made quiescent and then allowed to migrate in collagen-coated Transwell filters in the absence or presence of 10 nM R1881. Migrated cells were scored by fluorescent microscope as described in Methods and data expressed as relative increase in number of migrated cells. Results from several independent experiments were collected and analyzed. Means and SEM are shown. ***n*** represents the number of experiments. The statistical significance of results was also evaluated by Student's *t* test. In cells challenged with 10 nM R1881, the difference in relative migration between cells transfected with FlnA siRNA and those transfected with control siRNA was significant (P<0.001). Also significant (P<0.005) was the difference in relative migration between cells transfected with control siRNA and left unstimulated or stimulated with 10 nM R1881. The inset in B shows the Western blot of lysate proteins with anti FlnA (upper) or anti tubulin (lower) antibodies. In **C** and **D**, AR-negative Cos-7 cells were transfected with wt human AR (AR wt) or its mutant (AR mut) unable to interact with FlnA. After transfection, the cells were made quiescent and then left untreated or treated for 5 min with 10 nM R1881. In **C**, lysate proteins containing similar amounts of AR or FlnA or tubulin (upper section) were immunoprecipitated using anti-FlnA antibody. Proteins in immune complexes were analyzed by Western blotting using the antibodies against the indicated proteins (lower section). In **D**, cell lysates were analyzed by immunoblotting using anti-P-Tyr 397 FAK (P-FAK), FAK (FAK), anti-P-Tyr 118 paxillin (P-Pax), or paxillin (pax) antibodies. Data were analyzed using the NIH Image J program and expressed as relative increase. Numbers in the lower portion of corresponding blots represent the increase in P-FAK or P-pax. Expression of wt AR or its mutant (AR mut) was verified by immunoblotting using the C-19 anti-AR antibody (upper section). In **E**, AR-negative MDA-MB231 cells on coverslips were transfected with the pSG5 empty plasmid or pSG5- wt AR (AR wt) or pSV1 mutant AR (AR mut). After transfection, the cells were made quiescent and then left unstimulated or stimulated for 18 h with 10 nM R1881. After *in vivo* pulse with bromodeoxyuridine (BrdU), DNA synthesis was analyzed by IF and calculated by the formula: percentage of BrdU-positive cells =  (No. of transfected BrdU-positive cells/No. of transfected cells) X 100. For each plasmid, data were derived from at least 500 transfected cells. Results of three independent experiments were averaged. Means and SEM are shown. ***n*** represents the number of experiments. The statistical significance of results was also evaluated by Student' *t* test. The difference in BrdU incorporation between cells transfected with wt hAR (wt) and those transfected with pSG5 empty vector stimulated with R1881 was significant (P<0.001). Also significant (P<0.001) was the difference in BrdU incorporation between cells transfected with mutant hAR (mut) and those transfected with pSG5 empty vector stimulated with R1881. Lysate proteins were also immunoblotted using the C-19 anti-AR antibody (upper inset in **E**). The levels of tubulin (tub) were also assessed as a loading control (lower inset in **E**).

To dissect the role of the AR/FlnA/integrin beta 1 complex in androgen action, we silenced FlnA or integrin beta 1 in NIH3T3 fibroblasts and analyzed the effect of each knockdown in androgen-induced Rac 1 activation. Fig. 9A shows that silencing of either FlnA or integrin beta 1 abolishes the androgen activation of Rac 1 analyzed by pull-down assay. The role of FlnA in androgen-induced cell migration was supported by experiments in panel B showing that FlnA knockdown (insert in panel B) almost completely abolishes the migratory phenotype of hormone-treated NIH3T3 fibroblasts.

The role of AR/FlnA association was further analyzed by ectopically expressing in Cos-7 cells an AR-deleted mutant, unable to interact with FlnA [9]. Fig. 9C (lower panels) confirms that regardless of hormonal stimulation this mutant does not undergo association with FlnA. In contrast, the wild-type AR clearly co-immunoprecipitates with FlnA upon 10 nM R1881 stimulation. Upper panels in C show that similar amounts of AR or FlnA or tubulin were loaded in the co-immunoprecipitation experiment. Using the same experimental conditions, we observed that the mutant AR fails to induce tyrosine phosphorylation of both FAK and paxillin upon 10 nM R1881 stimulation (Fig. 9D). In cells transfected with wt AR, R1881 triggers FAK activation and increases paxillin tyrosine phosphorylation. Albeit similar amounts of total FAK were loaded, an increase in P-Tyr FAK was observed in lysates from untreated Cos cells ectopically expressing the mutant AR when compared with untreated cell expressing wt AR. These findings might be related to the scaffolding role of FlnA. Thus, the failure of mutant AR to interact with FlnA could fully channel FlnA towards integrin beta 1 or other proteins (eg Trio, ezrin). As a consequence, the binding of integrin beta 1 to FAK or the interaction of these proteins with the FAK-FERM domain could be facilitated. In such a way, allosteric or conformational restraints limiting FAK activity might be removed and FAK activation enhanced [19].

Although unable to interact with FlnA, the mutant AR still mediates androgen-induced DNA synthesis as shown by BrdU incorporation analysis of AR-negative human mammary cancer-derived MDA-MB231 cells ectopically expressing this mutant (Fig. 9E). In the same experiment, we analyzed for comparison BrdU incorporation induced by androgen stimulation of MDA-MB231 cells ectopically expressing wt AR (Fig. 9E).

Collectively, our findings show that androgen-triggered AR/FlnA/integrin beta 1 complex assembly controls FAK and Rac activation as well as migration in NIH3T3 fibroblasts, whereas it does not affect DNA synthesis.

## Discussion

In different cell types androgens stimulate signaling pathway activity usually triggered by growth factors, cytokines and extra cellular matrix (ECM). This ‘non-genomic’ action occurs within seconds or minutes and is independent of steroid receptor transcriptional activity [25]. Signaling activation by androgens as well as by other sex steroids controls cell growth in mouse models of mammary and prostate tumorigenesis and cell cycle progression, epigenetic modifications, steroid receptor (SR) localization as well as cytoskeleton changes in various cell types [4], [26]–[39].

In this paper we report a novel non-genomic action of classical AR. In NIH3T3 fibroblasts and human fibrosarcoma HT1080 cells, a physiological concentration of androgen promotes migration. Stimulation of cell migration is also observed in NIH3T3 fibroblasts triggered by DHT (not shown) and androgen specificity is shown by the failure of estradiol or progestins to trigger the migratory phenotype of NIH3T3 fibroblasts. In fact, these cells harbor low levels of classical AR, but do not express ERs or PR (present data) and [4].

We analyzed the molecular mechanism underlying androgen-induced cell migration in mouse NIH3T3 fibroblasts. In these cells, R1881 rapidly triggers assembly of a complex made up of AR, Fln A and integrin beta1 at a concentration stimulating migration (10 nM), but not at a concentration that poorly drives migration whereas actively induces G1-S transition (1 pM) [4]. siRNA interference experiments show that the AR/FlnA/integrin beta 1 complex controls androgen-induced cell migration in NIH3T3 fibroblasts. In apparent contrast with our results, previous findings have reported that stimulation of prostate cancer cells with testosterone-BSA reduces their migration rate. This inhibitory effect is, however, insensitive to three different anti-androgens and has been attributed to a membrane AR (mAR) [37], [40]. Our previous [4] and present findings show that AR expressed in fibroblasts is the classical AR since its DNA sequence contains the predicted sequence of mouse AR [4], it is sensitive to the anti-androgen Casodex and, more importantly it can be silenced by targeting the AR mRNA with a consequent loss of the androgen-induced migratory phenotype. Further, AR expressed in NIH3T3 fibroblasts does not seem to be localized at cell membrane, as shown by confocal microscopy analysis. Thus, on the basis of different criteria it appears to be distinct from AR identified on plasma membranes of prostate LNCaP cells. The opposite action of classical AR and mAR refractory to anti-androgens on cell migration might also implicate a negative reciprocal cross talk in cells expressing both receptors, such as LNCaP.

Our findings indicate that AR is a ‘hub protein’ that links androgen signaling to FlnA and integrin beta 1 in NIH3T3 fibroblasts. Results obtained using FlnA and integrin beta 1 silencing support an unexpected model of ’inside-out’ integrin activation by steroid hormones. Noteworthy, integrin beta 1 is closely related to primordial germ cell (PGC) migration and mouse PGC lacking integrin beta 1 fail to migrate normally to the gonads [41]. This phenotype is reminiscent of male androgen receptor knockout (ARKO) mice, which exhibits severe developmental defects and spermatogenesis arrest [42]. Thus, integrin beta 1 functions might intersect androgen signaling during embryogenesis. The AR/FlnA interaction here observed is an important part of this process. A calpain-generated FlnA fragment has been previously identified as an AR co-regulator in cell nuclei [9], [10]. Confocal microscopy analysis here presented reveals a novel intracellular localization of AR/FlnA complex that impacts androgen-induced cell motility. These findings indicate that the intracellular localization of this complex dictates androgen responses. Thus, the AR/FlnA complex controls motility when poised close to the signaling effectors at cytoplasm (present report), while it helps the transcriptional machinery in nuclei [9], [10]. The recent observation that high levels of cytoplasmic FlnA are a hallmark of metastatic prostate cancer [12] supports the hypothesis that AR/FlnA complex detected upon androgen stimulation in cytoplasm of embryonic fibroblasts might be recapitulated during cancer progression and invasiveness. The behavior of highly metastatic human fibrosarcoma HT1080 cells, which still respond to androgen with increased motility, favors this hypothesis.

We have shown that 10 nM R1881 induces FAK auto-phosphorylation and tyrosine phosphorylation of paxillin at tyrosine 118. In contrast, weak FAK activation is observed at suboptimal R1881 concentration (1 pM), which poorly stimulates cell migration. Knockdown experiments of FlnA and integrin beta 1 reveal that both FAK activation and tyrosine phosphorylation of paxillin are controlled by the upstream AR/FlnA/integrin beta 1 complex induced by androgens. In addition, findings in FAK-null fibroblasts show that androgen-induced cell motility requires FAK. These results support the role of FAK in non-genomic signaling of steroids leading to cell migration and invasiveness. Rapid activation of FAK by sex steroids has been previously reported in breast cancer T47D [30] as well as MCF-7 cells [32], and a correlation between FAK activation and breast cancer metastasis has been described [43].

The focal adhesion scaffold protein paxillin mediates interaction between integrins and FAK, and undergoes tyrosine phosphorylation through a FAK- or Src-mediated mechanism [18]. Findings in FAK-/- fibroblasts show that androgen-induced phosphorylation of paxillin at tyrosine 118 depends on FAK. Nevertheless, this does not exclude the possibility that Src is involved in this process, since paxillin can be phosphorylated in additional tyrosine residues [18] and FAK-null fibroblasts usually exhibit reduced Src activity [19]. Interactions of FAK, Src, p130CAS and paxillin promote focal contact/focal adhesion turnover at the leading edge in fibroblasts [44]. Recruitment and activation of Src, FAK and p130CAS by ligand-coupled steroid receptors has been described in different cell types [32], [34], [35], [45]–[47] and has been correlated to proliferative and survival effects of steroids in breast and prostate cancer cells [26], [35], [47].

Rho family proteins control cytoskeleton dynamics and cell motility [20]. Small GTPases and their downstream effectors have been implicated in steroid action *in vitro* and *in vivo* [4], [5], [14], [15], [29], [30], [37]. The present findings causally correlate androgen activation of Rac 1 to the upstream AR/FlnA/integrin beta 1 complex, further pointing to the key role of this complex in hormonal action. As regards Rac activation, it should be noted that FlnA directly interacts with Trio, a guanine exchange factor (GEF) for Rac and Rho G [48]. FlnA also interacts with Rac and Fil-GAP for Rac [22], [49]. These interactions might contribute to Rac regulation by androgens. Nonetheless, the finding that integrin beta 1 silencing also abolishes Rac activation (Fig. 9A) suggests that FlnA requires integrin beta 1 to activate Rac upon androgen challenging of the cells. Our findings in FAK-null fibroblasts or use of the Rac inhibitor EHT show that androgen-induced FAK or Rac activation are independent of each other. The absence or inhibition of either FAK or Rac, even induces a stronger activation of the other. Such a forward loop could be explained by the absence of FAK association with Rho-GTPases regulators in FAK-null fibroblasts. This might enable the increase in androgen-induced Rac activation observed in these cells. Conversely, in cells treated with the Rac inhibitor EHT, the Trio-GEF might be channeled towards the FERM domain of FAK, thus removing conformational restraints limiting FAK activity. The present findings indicate that activity of both FAK and Rac is mutually and discretely regulated by the upstream AR/FlnA/ntegrin beta 1 complex. This control ensures a balance between FAK-regulated cell adhesion and Rac-induced cytoskeleton changes, so that cells can modulate their migration rate.

Results with HT1080 cells call for additional comments. Epidemiological and clinical studies suggest that age, sex and hormonal milieu exert an important influence on the natural history of human mesenchymal tumors. SRs, including AR, have been previously detected in a large set of human soft tissue sarcomas of different histological origin [50]. Nevertheless, HT1080 cells have been considered AR-negative [5], [51]. Our findings obtained using two different anti-AR antibodies as well as the inhibition of cell motility by the anti-androgen Casodex reveal that HT1080 cells harbor low levels of classical AR that mediate androgen-induced migration but are unable to activate gene transcription, as assessed by gene reporter assay. Consistent with these latter findings, it has been previously reported that androgens do not activate tissue plasminogen activator-CAT reporter construct transiently transfected in HT1080 cells [51]. Thus, in HT1080 and NIH3T3 cells, as well as in other non-reproductive cells, the very low amount of steroid receptors (eg AR, PR and ER beta) is responsible for their failure to activate gene transcription (present report) and [4], [52]. However, this low amount of SRs efficiently activates signaling pathways upon steroid stimulation. By over-expressing SRs in NIH3T3 [4], Cos [4], rat uterine stromal [52] and HT1080 cells, gene transcription can be rescued in the presence of a rapid, transient and vigorous stimulation of signaling pathways. In contrast, low amounts of AR are sufficient to activate signaling transducing effectors [4]. Thus, differential responses may be achieved in different cells depending on their SR content. In cells (eg breast and prostate-derived cancer cells) expressing nano-molar amounts of SRs, these undergo significant dimerization in the presence of ligand and respond to nano-molar concentrations of steroid hormones with optimal nuclear translocation and transcriptional activation. In contrast, in cells expressing sub-nano-molar concentrations of SRs, the majority of receptor molecules are in the monomeric form in the absence of ligand. In this form, SRs are prevalently localized in the cytoplasm, where they activate signaling cascades.

Androgen-induced cytoskeleton changes have been previously observed in HT1080 cells engineered to stably over-express AR (HT-AR1 cells) and these changes have been correlated to the transcriptional up-regulation of Rho B mediated by AR [5]. It is unlikely that this transcriptional control occurs in our experimental setting, since AR expressed in HT1080 cells is devoid of transcriptional activity. Furthermore, androgens fail to activate Rho in NIH3T3 fibroblasts (Fig. S4, A). In HT1080, as in NIH3T3 cells, Rac inhibition impairs androgen-induced cell motility, suggesting that the same signaling effectors are engaged by androgens in both mesenchymal and mesenchimal transformed cells. Thus, HT1080 cells represent a new model of hormone-responsive cells that will prove useful in dissecting the role of androgen signaling in human cancer metastasis.

In summary, this report reveals new facets of androgen biology. It shows for the first time that classical AR stimulates cell migration and analyzes the molecular mechanism underlying this action. Our findings identify the androgen-induced AR/FlnA/integrin beta 1 complex as being the initial event triggering cell motility. By further exploiting this complex in a wide range of human androgen-dependent cancers, it should be possible to gain valuable insights into the role of androgens in human cancer metastasis. Lastly, steroid receptor sequences interacting with signaling effectors or scaffolds represent potential therapeutic targets to specifically inhibit signaling effectors involved in hormone action. This is, indeed, the case of ER alpha and AR sequence interacting with Src [36], [38].

## Materials and Methods

### Constructs

cDNA encoding the wild-type hAR was in pSG5 [53]. The mutant AR (Δ622-670 hAR) was in psV1 expression vector [9]. The 3416 construct, containing four copies of the wild-type *slp*-HRE2 (5′-TGGTCAgccAGTTCT-3′) and the 3424 construct (5′-TGGACAgccAGTTCT-3′) were cloned in the Nh*e*I site in pTK-TATA-Luc [54].

### Cell culture, siRNA, transfection and transactivation assays

FAK-/- and FAK+/+ fibroblasts (ATCC) were cultured according to the manufacturer's instructions and made quiescent as reported [4]. FAK-null fibroblasts were made quiescent by 12 h of serum starvation. Human fibrosarcoma HT1080 and mouse 3T3-L1 cells were cultured in Dulbecco's modified Eagle medium (DMEM) supplemented with 10% fetal calf serum (FCS), 100 U/ml penicillin and 100 microg/ml streptomycin, and maintained at 37°C in humidified 5% CO_2_ atmosphere. The cells were made quiescent by serum starvation (0.5% charcoal-stripped FCS) for 18 h. Mouse embryo NIH3T3 fibroblasts, Cos-7, LNCaP and MDA-MB231 cells were cultured and made quiescent as described [4], [26], [35]. Cos-7 and MDA-MB231 cells were transfected with 1 microg of each purified plasmid, using the Superfect reagent (Qiagen, GMbH). After 24 h, transfected cells were made quiescent and used. For integrin beta 1 siRNA, a pool of 4 target-specific 20–25 nt siRNAs (Santa Cruz) was used. FlnA silencing was performed using a pool of 3 target-specific 20–25 nt siRNAs (Santa Cruz). For AR siRNA, a pool of 4 target-specific 20–25 nt si RNAs (Santa Cruz) was used. Non-targeting siRNAs, containing a scrambled sequence, was from Santa Cruz. siRNA was transfected using Lipofectamine™ 2000 (Gibco). After transfection, the cells were made quiescent for 48 h and then used. Transactivation assay in subconfluent HT1080 cells was performed in phenol red–free DMEM containing 10% charcoal-stripped serum. Cells were transfected by Superfect with 2 microg of 3416-pTK-TATA- Luc or 3424-pTK-TATA-Luc construct, alone or with 1 µg pSG5- hAR-expressing plasmid. After 24 h, transfected cells were left unstimulated or stimulated with 10 nM R1881 (Perkin Elmer) for 18 h. Luciferase activity from lysates was measured using a luciferase assay system (Promega) and values corrected using CH110-expressed-beta-galactosidase activity (GE Healthcare).

### Wound scratch analysis, Transwell assay and time-lapse video microscopy

For wound scratch assay, quiescent cell monolayers at confluence were wounded using sterile pipette tips, and then washed in PBS. To avoid proliferation, the cells were treated with cytosine arabinoside (at 100 microM; Sigma) and then left unstimulated or stimulated for different times with the indicated concentrations of R1881. Casodex was from Astra Zeneca. Estradiol was from Sigma and the progestin R5020 from Roussel-Uclaf. The Rac 1 inhibitor EHT 1864 (Sigma) [55] was added 4 h before hormonal stimulation. Fields were analyzed with DMIRB inverted microscope (Leica) using N-Plan 10x objective (Leica). Contrast-phase images were captured using a DC200 camera (Leica) and acquired using IMI1000 (Leica) software. Transwell assay was performed using uncoated or collagen- (Type I from rat-tail at 100 mg/ml; BD Biosciences) coated Transwell chamber system with 8 microm pore polycarbonate membrane (Nunc). NIH3T3 cells were plated in the upper chamber at 2×10^4^ per well in 200 microl of phenol red-free DMEM containing 0.5% BSA. For AR and FlnA siRNA studies, NIH3T3 cells were co-transfected with siRNA Alexa Fluor 488 to help identify transfected cells. Cells were allowed to migrate for 6 h in a humidified chamber at 37°C with 5% CO_2_ in the absence or presence of the indicated compounds. Cells on the upper side were then detached. In NIH3T3 fibroblasts, cells on the underside were fixed in 4% paraformaldehyde for 15 min and stained with Hoechst 33258 for 10 min. In siRNA Alexa Fluor 488-transfected fibroblasts, the cells were fixed in 4% paraformaldehyde for 15 min. The cells were finally counted with a DMBL (Leica) fluorescent microscope using HCPL Fluotar 20x objective in ten random microscopic fields. The 2D random migration assay was carried out by seeding growing NIH3T3 fibroblasts onto 12 well plates at 2.5×10^4^ cells/well. Cells were made quiescent, and then left unstimulated or stimulated with R1881. Cytosine arabinoside (at 50 microM) was added to each sample. Cells were followed for 24 h and images from different samples were acquired every 10 min using the Zeiss Cell Observer System, made up of a motorized inverted microscope (Axiovert 200M), an incubator chamber for observation of living cells and a digital camera (Axiocam H/R). The obtained movies were analyzed as described [56], using the MotoCell semi-automatic tool. Quantitative analysis of migration was performed using the statistical analysis tool available within MotoCell. Average speed was calculated by averaging the length of cell displacements at each 40 min time step. For each cell, linearity was calculated as the ratio between net displacement and total path length.

### Cytoskeleton changes and DNA synthesis analysis

Cytoskeleton analysis in quiescent NIH3T3 cells was performed as reported [4], using Texas red-labeled phalloidin (Sigma). EHT 1864 was added 2 h before hormonal stimulation. BrdU incorporation in transfected MDA-MB231 cells was analyzed by immunofluorescence [26], using diluted (1∶50 in PBS) mouse monoclonal anti-BrdU antibody (clone BU-1, from GE Healthcare). Mouse antibody was detected using diluted (1∶200 in PBS) Texas red-conjugated goat anti-mouse antibody (Jackson Laboratories).

### Immunofluorescence and confocal microscopy

Cells on coverslips were fixed and permeabilized as described [26]. Endogenous AR in LNCaP cells and wild-type hAR ectopically expressed in Cos cells were visualized as described, using the rabbit polyclonal anti-C19 antibody [4]. In NIH3T3 and HT1080 cells, AR was visualized using diluted (1∶100 in PBS) rabbit polyclonal anti-AR antibody (Ab-2, Neo-Markers). Rabbit antibody was detected using diluted (1∶200 in PBS containing 0.2% bovine serum albumin) anti-rabbit fluorescein-conjugated antibodies (Jackson Laboratories). FlnA was detected using diluted (1∶40 in PBS) goat polyclonal anti-FlnA antibody (Ab11074; Abcam). Goat antibody was detected using diluted (1∶100 in PBS) rabbit anti-goat Texas red-conjugated antibody (Abcam). Total P-Tyr was detected using diluted (1∶100 in PBS) mouse monoclonal anti-P-Tyr antibody (clone 4G10, Upstate Biotechnology). Diluted (1∶200 in PBS) anti-mouse fluorescein isothiocyanate-conjugated antibody (Calbiochem) was added to detect the primary mouse antibodies. Coverslips were finally stained with Hoechst 33258, inverted and mounted in Mowiol (Calbiochem). Fields were analyzed with a DMBL Leica (Leica) fluorescent microscope using HCX PL Apo 63x oil and HCX PL Fluotar 100x oil objectives. Images were captured using DC480 camera (Leica) and acquired using FW4000 (Leica) software. Confocal microscopy analysis was performed using a Zeiss LSM 510 laser scanning confocal microscope as reported [33]. For AR/FlnA co-localization analysis, we used Argon/2 (458, 477, 488, 514 nanometers) and HeNe1 (543 nanometers) excitation lasers, which were separately switched on to reduce cross talk between the two fluorochromes. The green and the red emissions were separated by a dichroic splitter (FT 560) and filtered (515-to 540-nm band-pass filter for green and >610-nm long pass filter for red emissions. A threshold was applied to the images to exclude about 99% of the signal found in control images. The weighted co-localization coefficient represents the sum of intensity of co-localizing pixels in channels 1 and 2 as compared to the overall sum of pixel intensities above threshold. This value could be 0 (no co-localization) or 1 (all pixels co-localize). Bright pixels contribute more than faint pixels. The co-localization coefficient represents the weighted co-localization coefficients of Ch1 (red) with respect to Ch2 (green) for each experiment [57]. The image collection periods and exposures were identical for the different experimental conditions.

### Lysates, immunoprecipitation, GTP-binding protein assay and immunoblotting

Lysates (at 2 mg/ml protein concentration) were prepared as described [34]. FlnA was immunoprecipitated using rabbit polyclonal anti-FlnA antibody (Cell Signaling). FAK was immunoprecipitated using mouse monoclonal anti-FAK antibody (clone 77; BD Biosciences). Mouse monoclonal anti-P-Tyr antibody (clone 4G10; Upstate Biotechnology) was used to immunoprecipitate P-Tyr phosphorylated proteins. Rac and Rho pull down assays were performed as described [4], using the Rac or the Rho activation kit (Upstate Biotechnology). AR was revealed using the rabbit polyclonal anti-AR antibody (Ab-2; NeoMarkers) or the rabbit polyclonal anti-AR antibodies (N-20 or C-19; Santa Cruz). The rabbit polyclonal anti-ERalpha antibody (543; Santa Cruz) was used to detect ERalpha. PR was detected using the mouse monoclonal anti-PR antibody (6A1; Cell Signaling). The rabbit polyclonal anti-FlnA antibody (Cell Signaling) was used to detect FlnA. Integrin beta 1 was detected using rabbit polyclonal anti-integrin beta 1 antibody (Chemicon International), whereas the rabbit polyclonal anti-integrin beta 3 (Santa Cruz) was used to detect integrin beta 3. Total FAK, P-Tyr 397, P-Tyr 925 FAK, total Akt and P-Ser 473 Akt were detected using appropriate antibodies (Cell Signaling). P-Tyr 118 paxillin was detected using rabbit polyclonal anti-P-Tyr 118 paxillin antibody (BD Biosciences). Mouse monoclonal anti-paxillin antibody (clone 349; BD Biosciences) was used to detect total paxillin. Rho A, Erk and P-Tyr 204 Erk were detected using appropriate antibodies (Santa Cruz). Immune-reactive proteins were revealed by the ECL detection system (from GE Healthcare).

## Supporting Information

Figure S1 **Estradiol and progestins do not affect fibroblast migration.** NIH3T3 fibroblasts do not express ERalpha or PR, as assessed by Western blot analysis of cell lysates using appropriate antibodies (Fig. S1, A). Western blot analysis of lysate proteins from breast cancer-derived cells (MCF-7 and T47D cells) is shown for comparison. Western blot analysis using the anti-tubulin antibody was performed as a loading control (tub). In agreement with findings in **A**, contrast-phase images in **B** show that quiescent NIH3T3 fibroblasts do not migrate in wound scratch assay upon stimulation with 10 nM of E_2_ or R5020. In contrast, the cells migrate upon 10 nM R1881 stimulation. The effect of suboptimal (1 pM) R1881 concentration on cell migration is negligible. Images are representative of two independent experiments, each performed in duplicate.
(TIF)

Figure S2 **Time course of androgen-induced cytoskeleton changes in NIH3T3 fibroblasts.** Quiescent NIH3T3 fibroblasts on coverslips were left unstimulated or stimulated for the indicated times with 10 nM R1881 and then analyzed by IF for F-actin. Images are representative of three independent experiments. Arrows mark the cytoskeleton changes (ruffles and protrusions) induced by androgen treatment of NIH3T3 cells. Scale bar, 5 microM.
(TIF)

Figure S3 **Estradiol and progestins do not affect FAK activation and paxillin tyrosine phosphorylation in NIH3T3 cells.** Quiescent NIH3T3 fibroblasts were left untreated or challenged for the indicated time with either 10 nM estradiol (E_2_; panel **A**) or 10 nM R5020 (panel **B**). Similar amounts of total FAK or paxillin (upper sections in **A** and **B**) were immunoprecipitated with anti-P-Tyr antibody. Proteins in immunocomplexes were immunoblotted with antibodies against P-FAK or P-paxillin (lower sections in **A** and **B**).
(TIF)

Figure S4 **Ten nM R1881 does not activate Rho or Akt or Erk in NIH3T3 fibroblasts.** Quiescent NIH3T3 cells were used. In **A**, cells were left untreated or treated for the indicated times with 10 nM R1881 or EGF (100 ng/ml). Lysate proteins were assayed for Rho activation in pull-down assay. Loaded (input) and eluted (Rho-GTP) proteins were analyzed by immunoblotting using the anti-Rho A antibody. In **B** and **C**, cells were left untreated or treated for 5 minutes with the indicated compounds (EGF was used at 100 ng/ml; R1881 was used at 1 pM or 10 nM). In **B**, lysate proteins were analyzed by immunoblotting using anti P-AKT (upper panel) or anti-AKT (lower panel) antibodies. In **C**, lysate proteins were analyzed by immunoblotting using anti-P-Erk (upper panel) or anti-Erk (lower panel) antibodies.
(TIF)

Figure S5 **Androgen induces nuclear translocation of AR in human prostate cancer-derived LNCaP cells and Cos cells ectopically expressing wild-type hAR.** Left panels: LNCaP cells on coverslips were used and made quiescent. Right panels: AR-negative Cos cells on coverslips were transiently transfected with wild-type hAR-encoding plasmid, then made quiescent. The cells were left unstimulated or stimulated for 1 h with 10 nM R1881 and then analyzed by IF for AR as described in Methods. AR intracellular distribution was analyzed by confocal microscopy (LNCaP cells) or immunofluorescence microscopy (Cos cells). For each cell line, several fields were analyzed and representative images from two independent experiments were captured and shown. Scale bars, 10 microM (LNCaP cells) or 5 microM (Cos cells).
(TIF)

Figure S6 **Ten nM R1881 does not trigger recruitment of integrin beta 3 to AR/FlnA complex in NIH3T3 fibroblasts.** Quiescent NIH3T3 cells were left untreated (basal) or treated for 5 min with 10 nM R1881 (R1881) in the absence or presence of 10 µM Casodex (Cx). Cell lysates were immunoblotted with antibody against FlnA (loading). Lysate proteins containing similar amounts of FlnA were immunoprecipitated with either control (ctrl) or anti-FlnA antibody. Proteins in immunocomplexes were analyzed by immunoblotting using antibodies against the indicated proteins.
(TIF)

Figure S7 **Ten nM R1881 does not induce AR/FAK co-localization in NIH3T3 fibroblasts.** Quiescent NIH3T3 cells on coverslips were treated for 5 min with 10 nM R1881. Cells on coverslips were visualized by IF for AR and FAK as described in Methods. Images captured by confocal microscope show the staining of AR (green) and FAK (red). Lower panel shows the merged image. Scale bar: 5 microM
(TIF)
