# Supplementary figures and images for: Androgen-Induced Cell Migration: Role of Androgen Receptor/Filamin A Association
Source: PLoS One. 2011 Feb 16;6(2):e17218. doi: 10.1371/journal.pone.0017218 (PMC3040221; doi:10.1371/journal.pone.0017218)

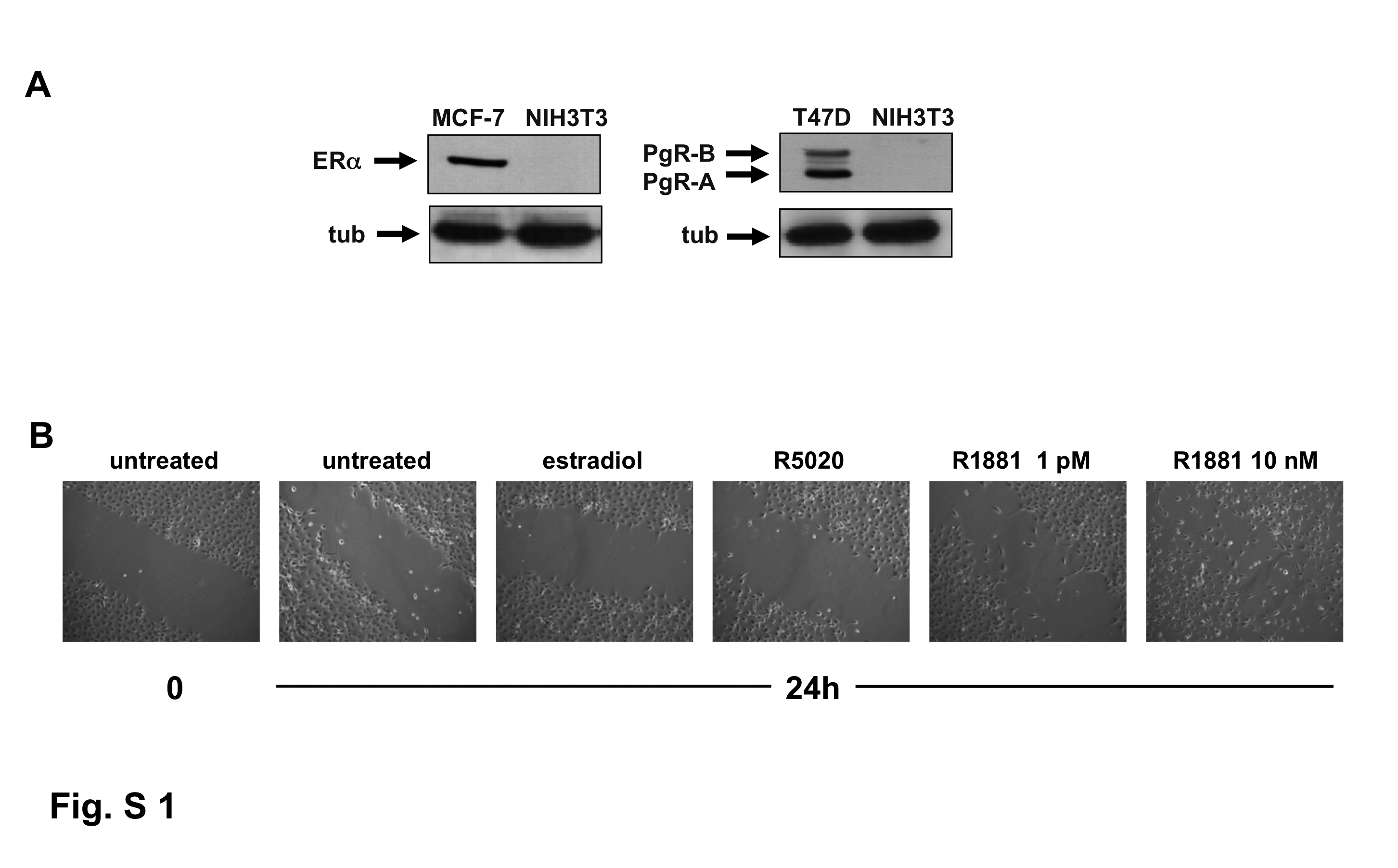

Supplement: Figure S1 — Estradiol and progestins do not affect fibroblast migration. NIH3T3 fibroblasts do not express ERalpha or PR, as assessed by Western blot analysis of cell lysates using appropriate antibodies (Fig. S1, A). Western blot analysis of lysate proteins from breast cancer-derived cells (MCF-7 and T47D cells) is shown for comparison. Western blot analysis using the anti-tubulin antibody was performed as a loading control (tub). In agreement with findings in A, contrast-phase images in B show that quiescent NIH3T3 fibroblasts do not migrate in wound scratch assay upon stimulation with 10 nM of E2 or R5020. In contrast, the cells migrate upon 10 nM R1881 stimulation. The effect of suboptimal (1 pM) R1881 concentration on cell migration is negligible. Images are representative of two independent experiments, each performed in duplicate. (TIF) [file pone.0017218.s001.tif]

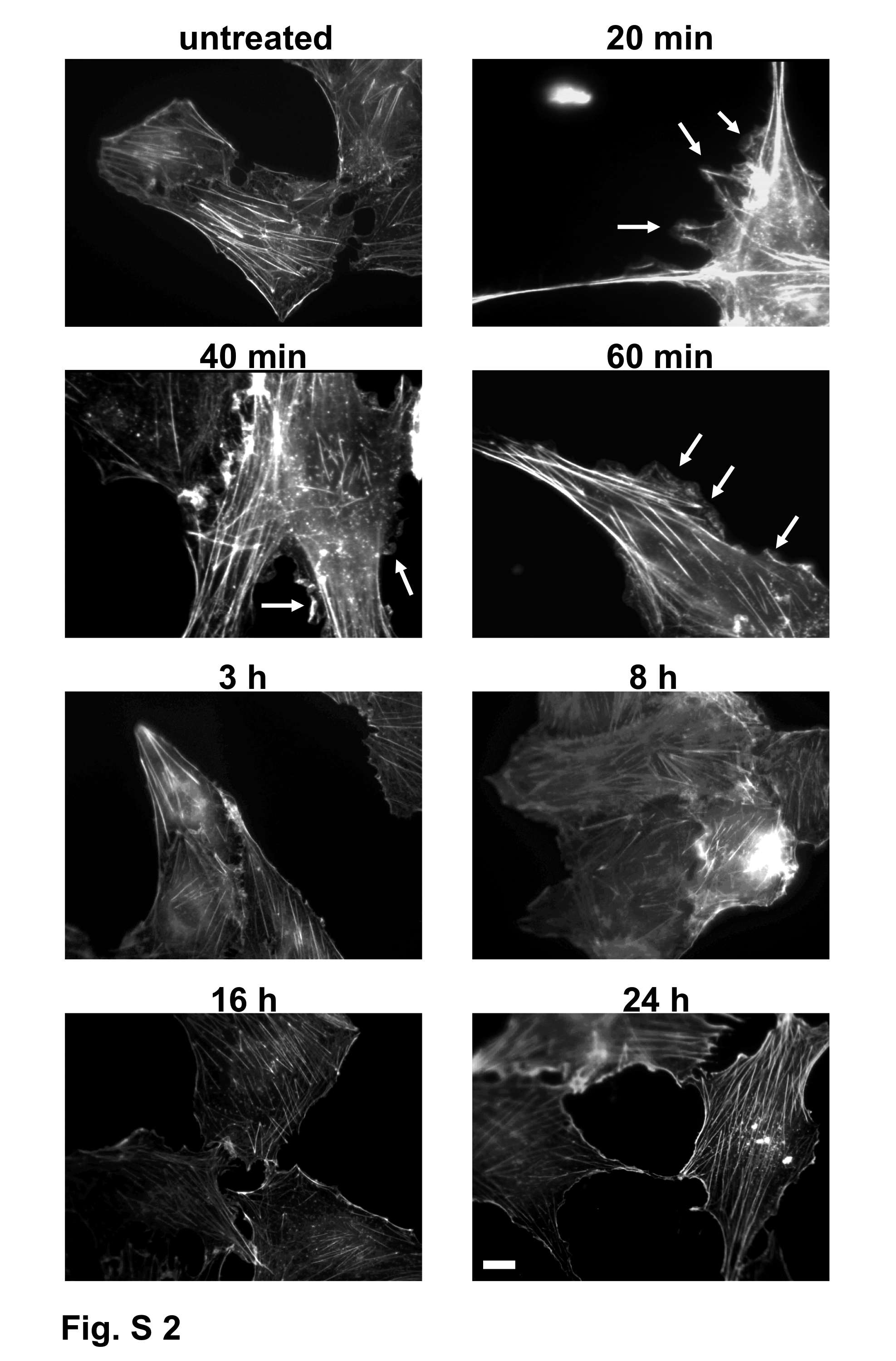

Supplement: Figure S2 — Time course of androgen-induced cytoskeleton changes in NIH3T3 fibroblasts. Quiescent NIH3T3 fibroblasts on coverslips were left unstimulated or stimulated for the indicated times with 10 nM R1881 and then analyzed by IF for F-actin. Images are representative of three independent experiments. Arrows mark the cytoskeleton changes (ruffles and protrusions) induced by androgen treatment of NIH3T3 cells. Scale bar, 5 microM. (TIF) [file pone.0017218.s002.tif]

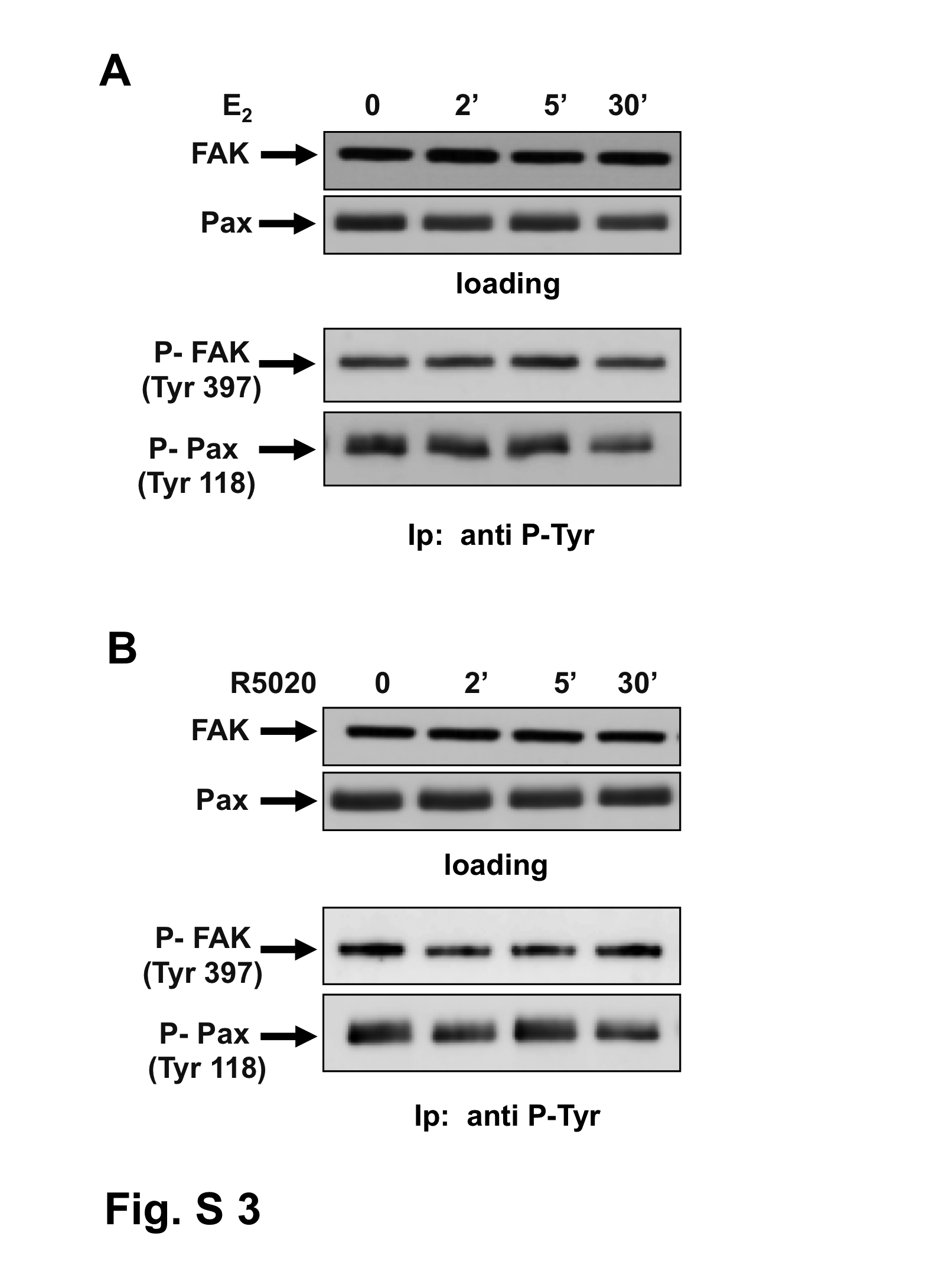

Supplement: Figure S3 — Estradiol and progestins do not affect FAK activation and paxillin tyrosine phosphorylation in NIH3T3 cells. Quiescent NIH3T3 fibroblasts were left untreated or challenged for the indicated time with either 10 nM estradiol (E2; panel A) or 10 nM R5020 (panel B). Similar amounts of total FAK or paxillin (upper sections in A and B) were immunoprecipitated with anti-P-Tyr antibody. Proteins in immunocomplexes were immunoblotted with antibodies against P-FAK or P-paxillin (lower sections in A and B). (TIF) [file pone.0017218.s003.tif]

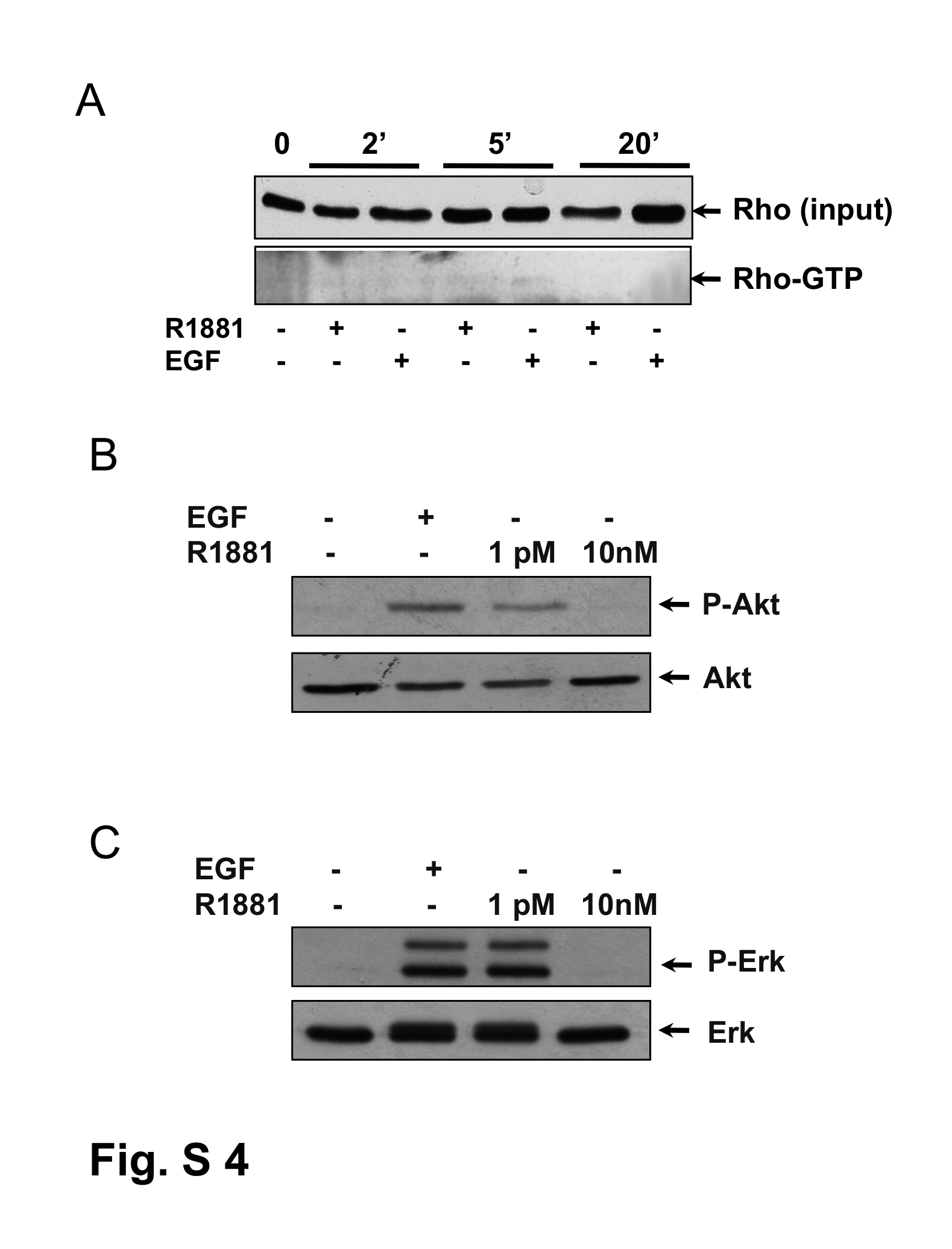

Supplement: Figure S4 — Ten nM R1881 does not activate Rho or Akt or Erk in NIH3T3 fibroblasts. Quiescent NIH3T3 cells were used. In A, cells were left untreated or treated for the indicated times with 10 nM R1881 or EGF (100 ng/ml). Lysate proteins were assayed for Rho activation in pull-down assay. Loaded (input) and eluted (Rho-GTP) proteins were analyzed by immunoblotting using the anti-Rho A antibody. In B and C, cells were left untreated or treated for 5 minutes with the indicated compounds (EGF was used at 100 ng/ml; R1881 was used at 1 pM or 10 nM). In B, lysate proteins were analyzed by immunoblotting using anti P-AKT (upper panel) or anti-AKT (lower panel) antibodies. In C, lysate proteins were analyzed by immunoblotting using anti-P-Erk (upper panel) or anti-Erk (lower panel) antibodies. (TIF) [file pone.0017218.s004.tif]

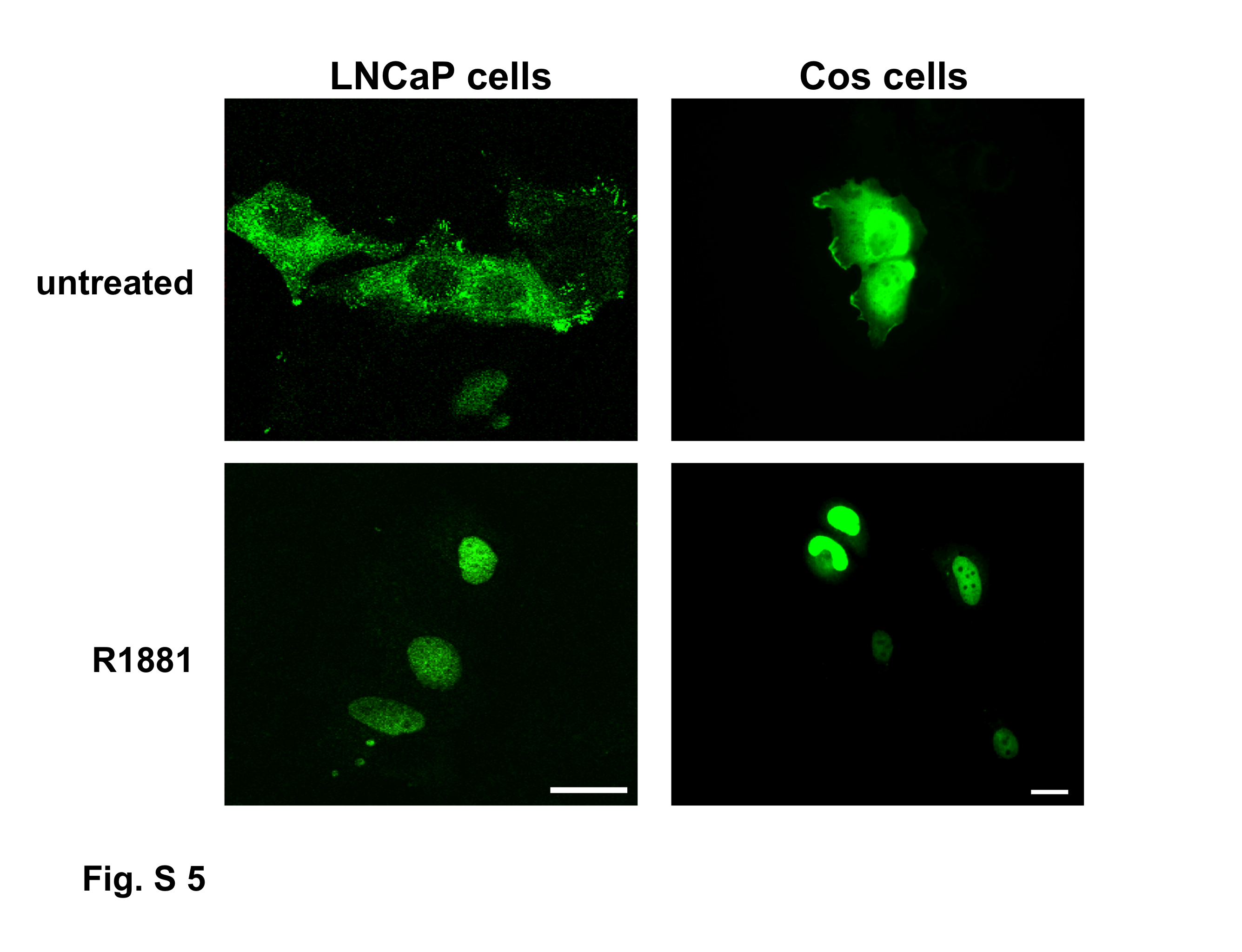

Supplement: Figure S5 — Androgen induces nuclear translocation of AR in human prostate cancer-derived LNCaP cells and Cos cells ectopically expressing wild-type hAR. Left panels: LNCaP cells on coverslips were used and made quiescent. Right panels: AR-negative Cos cells on coverslips were transiently transfected with wild-type hAR-encoding plasmid, then made quiescent. The cells were left unstimulated or stimulated for 1 h with 10 nM R1881 and then analyzed by IF for AR as described in Methods. AR intracellular distribution was analyzed by confocal microscopy (LNCaP cells) or immunofluorescence microscopy (Cos cells). For each cell line, several fields were analyzed and representative images from two independent experiments were captured and shown. Scale bars, 10 microM (LNCaP cells) or 5 microM (Cos cells). (TIF) [file pone.0017218.s005.tif]

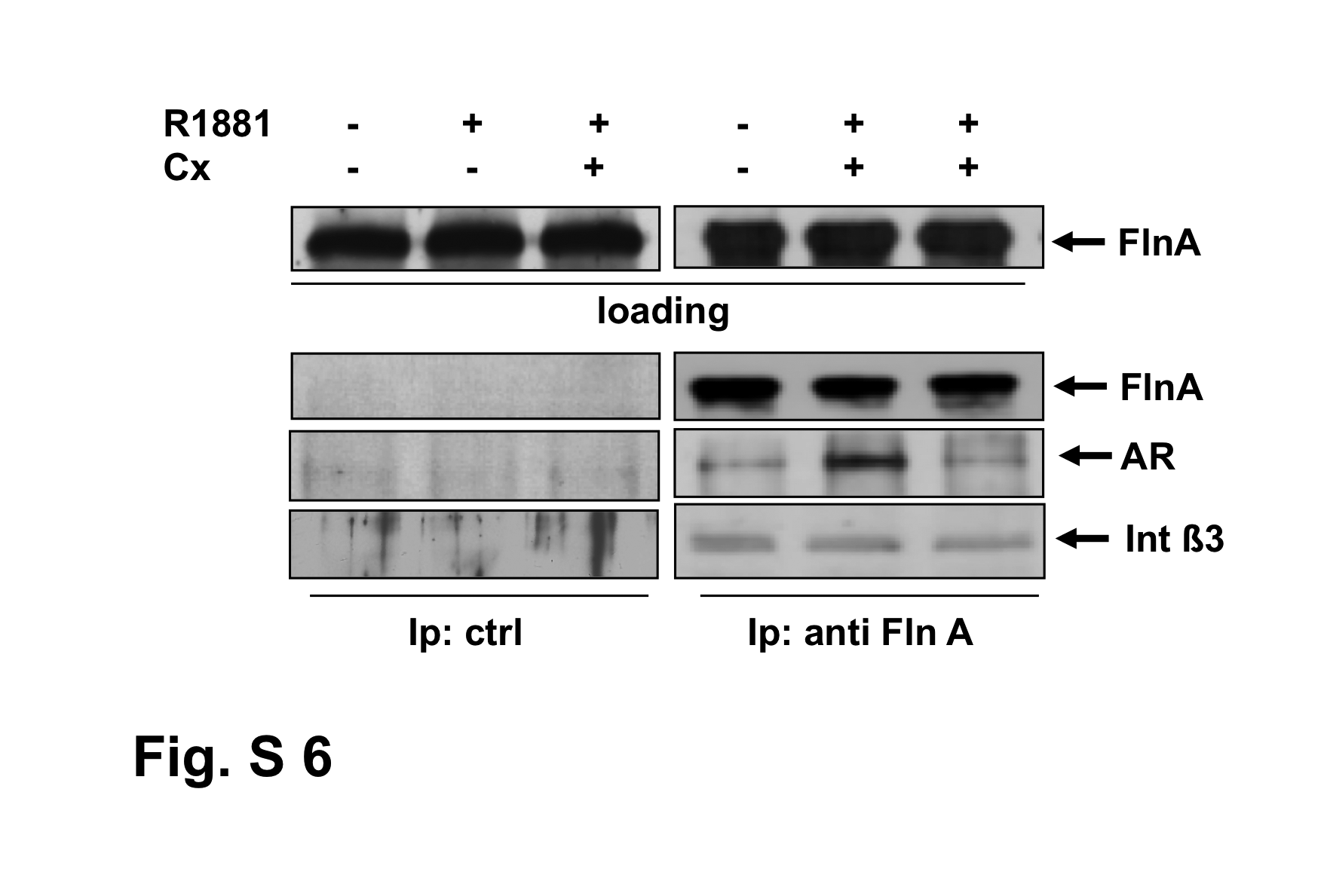

Supplement: Figure S6 — Ten nM R1881 does not trigger recruitment of integrin beta 3 to AR/FlnA complex in NIH3T3 fibroblasts. Quiescent NIH3T3 cells were left untreated (basal) or treated for 5 min with 10 nM R1881 (R1881) in the absence or presence of 10 µM Casodex (Cx). Cell lysates were immunoblotted with antibody against FlnA (loading). Lysate proteins containing similar amounts of FlnA were immunoprecipitated with either control (ctrl) or anti-FlnA antibody. Proteins in immunocomplexes were analyzed by immunoblotting using antibodies against the indicated proteins. (TIF) [file pone.0017218.s006.tif]

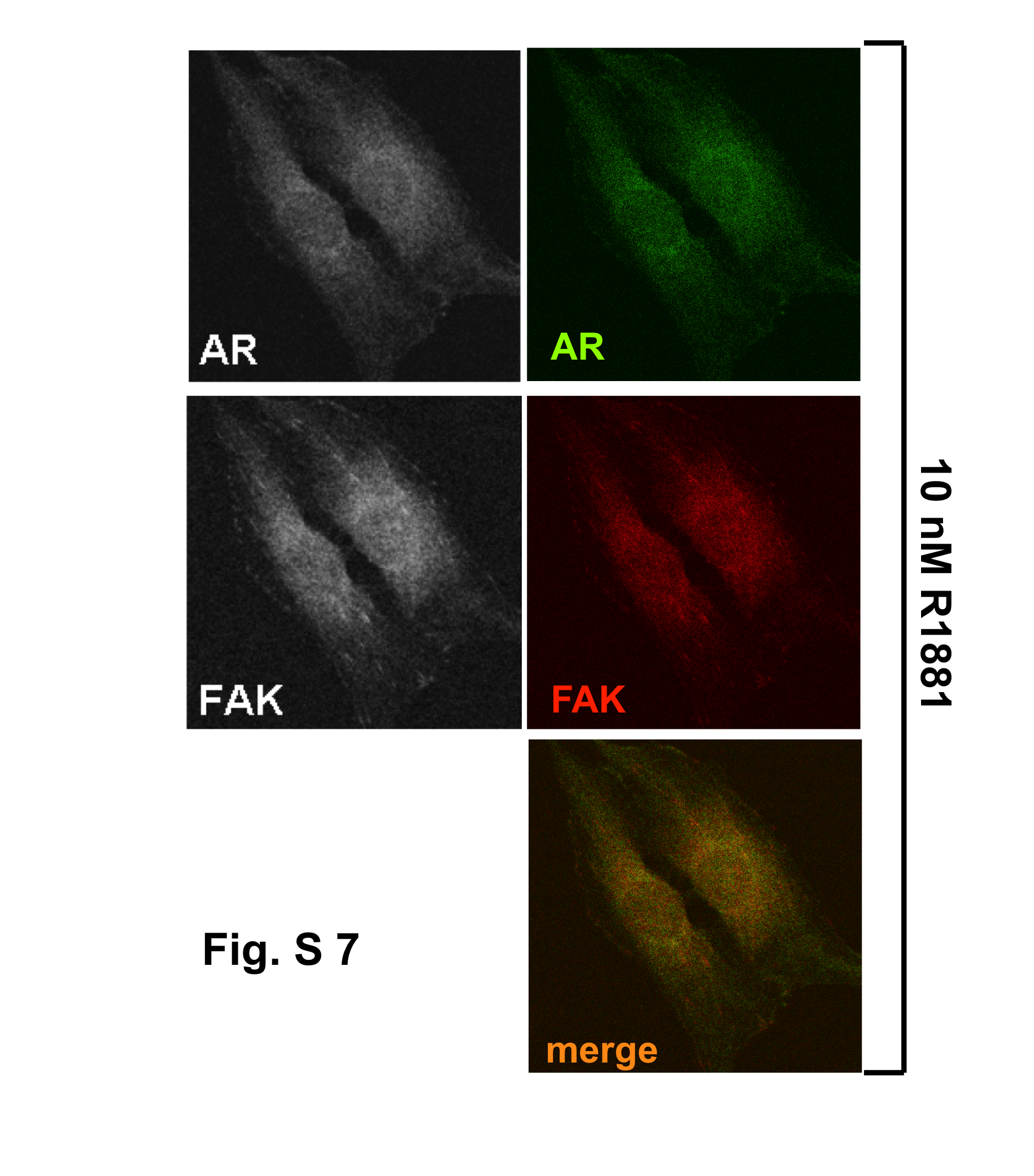

Supplement: Figure S7 — Ten nM R1881 does not induce AR/FAK co-localization in NIH3T3 fibroblasts. Quiescent NIH3T3 cells on coverslips were treated for 5 min with 10 nM R1881. Cells on coverslips were visualized by IF for AR and FAK as described in Methods. Images captured by confocal microscope show the staining of AR (green) and FAK (red). Lower panel shows the merged image. Scale bar: 5 microM (TIF) [file pone.0017218.s007.tif]
